# Genomic Expression Analysis Reveals Strategies of *Burkholderia cenocepacia* to Adapt to Cystic Fibrosis Patients' Airways and Antimicrobial Therapy

**DOI:** 10.1371/journal.pone.0028831

**Published:** 2011-12-21

**Authors:** Nuno P. Mira, Andreia Madeira, Ana Sílvia Moreira, Carla P. Coutinho, Isabel Sá-Correia

**Affiliations:** 1 IBB, Institute for Biotechnology and Bioengineering, Center for Biological and Chemical Engineering, Instituto Superior Técnico, Technical University of Lisbon, Lisbon, Portugal; 2 Department of Bioengineering, Instituto Superior Técnico, Technical University of Lisbon, Lisbon, Portugal; Louisiana State University and A & M College, United States of America

## Abstract

Pulmonary colonization of cystic fibrosis (CF) patients with *Burkholderia cenocepacia* or other bacteria of the *Burkholderia cepacia* complex (Bcc) is associated with worse prognosis and increased risk of death. During colonization, the bacteria may evolve under the stressing selection pressures exerted in the CF lung, in particular, those resulting from challenges of the host immune defenses, antimicrobial therapy, nutrient availability and oxygen limitation. Understanding the adaptive mechanisms that promote successful colonization and long-term survival of *B. cenocepacia* in the CF lung is essential for an improved therapeutic outcome of chronic infections. To get mechanistic insights into these adaptive strategies a transcriptomic analysis, based on DNA microarrays, was explored in this study. The genomic expression levels in two clonal variants isolated during long-term colonization of a CF patient who died from the cepacia syndrome were compared. One of the isolates examined, IST439, is the first *B. cenocepacia* isolate retrieved from the patient and the other isolate, IST4113, was obtained three years later and is more resistant to different classes of antimicrobials. Approximately 1000 genes were found to be differently expressed in the two clonal variants reflecting a marked reprogramming of genomic expression. The up-regulated genes in IST4113 include those involved in translation, iron uptake (in particular, in ornibactin biosynthesis), efflux of drugs and in adhesion to epithelial lung tissue and to mucin. Alterations related with adaptation to the nutritional environment of the CF lung and to an oxygen-limited environment are also suggested to be a key feature of transcriptional reprogramming occurring during long-term colonization, antibiotic therapy and the progression of the disease.

## Introduction

The *Burkholderia cepacia* complex (Bcc) is a heterogeneous group of bacteria comprising at least 17 closely related species that are ubiquitous, metabolically versatile and can cause chronic opportunistic infections in immunocompromised patients and in patients with cystic fibrosis (CF) [1]. In general, these bacteria lead to long-term colonization and to a more rapid decline in lung function of these patients and in some cases to the development of a fatal necrotizing pneumonia, accompanied by septicemia, known as the “cepacia syndrome”[1]. Bcc bacteria are often resistant to the most known clinically used antibiotics [1], [2], [3]. This trait and the ability to develop high-level resistance during antibiotic treatment and to adapt and resist to other adverse environmental conditions severely hinders the effective treatment of respiratory infections rendering their eradication from the CF lung very difficult, if not virtually impossible [1], [2]. During chronic colonization of the airways of CF patients, Bcc bacteria experience changing selection pressures, in particular those resulting from challenges of the immune defenses, antimicrobial therapy, nutrient availability and oxygen limitation [4]. The adaptive responses occurring in clinical isolates of *Pseudomonas aeruginosa*, another major respiratory pathogen of CF patients [5], have been on the focus of recent studies [6], [7], [8], [9], [10], [11], [12] but equivalent studies on Bcc bacteria are still lacking. Based on the collection of Bcc isolates gathered since 1995, as the result of systematic and longitudinal epidemiological surveys carried out by our group for the past 16 years of the respiratory infections of CF patients by Bcc followed at the major Portuguese CF Center, in Santa Maria Hospital (HSM) in Lisbon [3], [13], [14], we have been trying to get clues on the adaptive strategies adopted by *B. cenocepacia* during chronic infection. Our analysis was based on extensive phenotypic, genotypic and genome-wide expression approaches focusing selected Bcc isolates obtained during chronic colonization of different patients [3,15,16 and unpublished results]. In particular, we performed the systematic assessment of a number of relevant phenotypic characteristics in the context of CF infections, of eleven serial isolates obtained from a CF patient colonized during three and a half years until the patient's death with the cepacia syndrome [15]. These isolates are indistinguishable based on the *recA*-RFLP and *Eco*RI ribopattern profiles [17] and belong to the same clonal Based Upon Related Sequence Types (BURST) group [15], consistent with the idea that the CF patient's lungs can be chronically colonized for years by one or a few lineages of *P. aeruginosa* and Bcc bacteria [17], [18], [19], [20]. This systematic phenotypic analysis suggested the occurrence of clonal expansion of *B. cenocepacia* during chronic lung infection presumably as the result of mutations and selective pressures occurring in the CF lung environment, in particular due to host immune defenses, antibiotic therapy and oxygen limitation, as proposed for *P. aeruginosa* [6], [7], [9], [10], [11], [19], [21].

The present study is dedicated to the comparison of the genomic expression, based on DNA microarrays, of two of these 11 sequential *B. cenocepacia* isolates: isolate IST439 that was the first *B. cenocepacia* isolate retrieved from the patient and thought to have initiated the infection with this bacterium, and isolate IST4113 that exhibits increased levels of resistance to different classes of antimicrobials and was obtained three years later, after a period of exacerbated pulmonary infection that compelled the patient to hospitalization and intravenous therapy with gentamicin and ceftazidime [3], [16]. The proteomes of these same isolates were quantitatively compared before, based on two-dimensional gel electrophoresis (2-DE) using DIGE technology (Difference Gel Electrophoresis) [16]. Proteins of the functional categories Energy metabolism, Translation, Iron uptake, Nucleotide synthesis and Protein folding and stabilization were more abundant in IST4113, compared to IST439, suggesting an increased protein synthesis, DNA repair activity, iron uptake capacity and stress resistance in isolate IST4113 [16]. The concentration of proteins related with peptidoglycan biosynthesis and the synthesis of membrane lipids and of lipopolysaccharide was also found to be different in the two clonal variants [16]. Since protein coverage based on the quantitative proteomic analysis performed is limited, the here described transcriptomic analysis complements and extends the indications obtained before enlightening the genome-wide adaptive strategies of *B. cenocepacia* to adapt to cystic fibrosis patients' airways and antibiotic therapy during long-term colonization.

## Results

### Comparative analysis of the transcriptomes of *B. cenocepacia* isolates IST439 and IST4113

To compare the transcriptomes of *Burkholderia cenocepacia* IST439, thought to have started the infection of a cystic fibrosis (CF) patient with *B. cenocepacia*, and the clonal variant IST4113, retrieved three years later and exhibiting an increased resistance to all the antimicrobials tested [3], [15], [16], total RNA was extracted from cells cultivated for 24 hours onto the surface of LB plates, at 37°C, to mimic bacterial growth during chronic colonization of the CF patient's lung. These same conditions were used before to quantitatively compare the proteomes of these two isolates [16]. This microarray analysis indicated that, using a 1.5-fold threshold value, 1024 genes are differently expressed in the two variants, 534 genes being up-regulated and 490 genes down-regulated in the IST4113 variant, compared to IST439. A selected sub-set of the differently expressed genes in the two variants is shown in Supplementary Table S1 while the complete list is provided in supplementary tables S2 and S3.

The 1024 genes found to have an altered transcription in IST439 and IST4113 are distributed through the three *B. cenocepacia* chromosomes, ranging from 5 to 9.3% of the coding sequences present in each chromosome (Table 1). Almost one half of our microarray dataset of genes differently expressed in IST439 and IST4113 (474 genes out of 1024) corresponds to genes located in chromosome 1, containing genetic information for central metabolism and other core cellular functions [22]. Only one of the genes differently expressed in the two clonal variants (*pBCA015*) was found to be located in the natural plasmid pBCJ2315 (Table 1). Three of the genes more actively transcribed in IST4113, compared to IST439, are located within the *cci* pathogenicity island, a conserved DNA region among *B. cenocepacia* epidemic strains and involved in infection and antibiotic resistance [22]. These three genes encode a putative ion transporter (*BCAM0238*), a N-acylhomoserine lactone-dependent regulatory protein (*cciR*) and a gene of unknown function (*BCAM0243)* [23]. No other gene located in the described genomic islands present in *B. cenocepacia* J2315 genome was found to be differently expressed in the clonal variants examined, consistent with the concept that *B. cenocepacia* pathoadaptation to CF lung relates with modifications occurring at the level of the inherent traits of the bacterium instead of being acquired through mobile genetic elements [24].

**Table 1:** Distribution of the genes differently transcribed (above or below a 1.5-fold) in IST439 and IST4113 through the *B. cenocepacia* J2315 genome.

	Up-regulated in IST4113	Down-regulated in IST4113
*Genes*	507	489
*Chromossome 1*	248 (7.0%)	226 (6.4%)
*Chromossome 2*	187 (6.5%)	224 (7.8%)
*Chromossome 3*	72 (9.3%)	39 (5.0%)
*Plasmid*	1	0
*tRNAs*	26	1
*Intergenic regions*	48	66

The genes found to be differently transcribed in IST439 and IST4113 variants, according with the microarray analysis carried out, were distributed through the 3 chromosomes and one plasmid (pBCJ2315) that compose the genome of *B. cenocepacia* J2315 strain [22]. Inside brackets it is indicated the percentage of genes differently transcribed in the two clonal variants, considering a total of 3537, 2849 and 776 coding sequences for chromosomes 1, 2 and 3, respectively [22]). The number of tRNAs, rRNAs and intergenic sequences (IGs) that exhibit altered transcript levels in the two variants is also indicated.

The microarray used in the transcriptomic analysis carried out includes probes for the detection of coding sequences (CDSs) of the J2315 epidemic strain, as well as for CDSs that are absent in this strain but present in the genome of two other sequenced strains, AU1054 (recovered from the blood of a CF patient) and HI2424 (a soil isolate) [24]. Eight genes specific of the AU1054 strain were found in our study among the genes differently expressed in the two variants. Three of these genes (*Bcen_1448*, *Bcen_1449* and *Bcen_2828*) are up-regulated in IST4113 while five of these genes (*Bcen_2651*, *Bcen_2885*, *Bcen_2886*, *Bcen_2900* and *Bcen_3131*) are more actively transcribed in IST439 (Supplementary Tables S2 and S3). Although these DNA regions are absent from J2315 genome, the two clonal variants examined, isolated in Portugal, belong to the same clonal complex as J2315 (complex 31) [15], [25] whereas strain AU1054 belongs to clonal complex 125 [25]. The transcript levels of *B. cenocepacia* HI2424-specific genes present in the DNA microarray used were identical in IST439 and IST4113.

**Figure 1:**
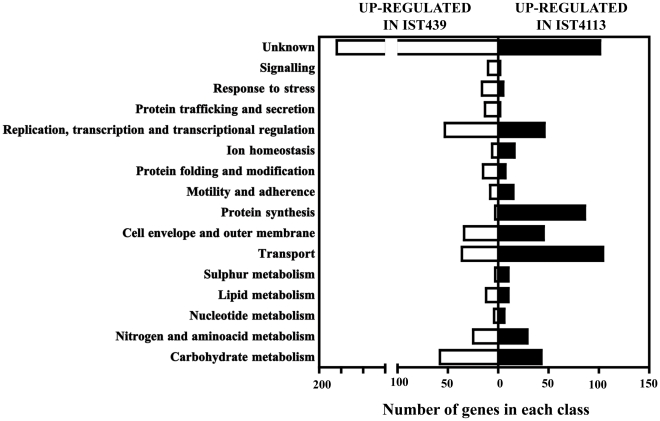
Clustering, based on biological function, of the genes found to be differently expressed in IST4113 (black bars) or in IST439 (white bars). The genes whose transcript level in the two variants varied above 1.5-fold were selected and grouped according to their biological function, based on the information available in the *Burkholderia* Genome database and in the KEGG Pathways Database.

The genes found to have an altered expression in IST439 and IST4113 were clustered according to their physiological function, using the information available in the *Burkholderia* Genome Database (http://www.burkholderia.com/) and in the KEGG pathways database (http://kegg.jp/kegg) (Fig. 1). Among the 1024 differently expressed genes, 311 do not have an attributed biological role and were clustered as “Unknown”. An enrichment of genes involved in protein synthesis, ion homeostasis (in particular, in iron uptake) and sulphur metabolism is evident among the genes with higher transcript levels in the IST4113 variant. The functional categories “Transport” and “Cell envelope and outer membrane” are also enriched in the dataset of genes transcriptionally activated in IST4113, although less significantly. The functional classes having a higher number of genes more actively transcribed in IST439 are “Replication, transcription and transcriptional regulation”, “Stress response”, “Signalling”, “Protein trafficking and secretion” and “Protein folding and modification” (Fig. 1). The genes involved in stress response found to be up-regulated in IST439 encode essentially proteins involved in response to oxidative stress, namely four enzymes involved in glutathione biosynthesis (*BCAL0639*, *BCAL1250*, *BCAM0590* and *BCAL3331*), a thioredoxin (*BCAL0463*), a peroxiredoxin (*BCAL2013*), the glutathione peroxidase Gpo and the peroxidase/catalase KatB (Supplementary Table S2). Moreover, the transcript level of ^70^σ-factor EcfA2, an RpoE-like regulator presumed to control the expression of genes required for response to heat and oxidative stress [26], in IST4113 cells are half of those registered in IST439 (Supplementary Table S3). Consistently, several components of the GroEL chaperone complex, also predicted to be regulated by the ^70^σ-factor EcfA2 [26], are down-regulated (in a range from 3.1 to 29-fold) in IST4113 cells (Supplementary Table S3). The other functional classes include a similar number of genes of the microarray dataset (Fig. 1) but, although clustered in the same class, the genes differently transcribed in the two variants have different functions (Supplementary Tables S2 and S3). For example, the genes clustered in the “Motility and adherence class” up-regulated in IST4113 are related with the biosynthesis and assembly of flagella (*flhB*, *flhF*, *fliK*, *fliJ*, *fliG*, *fliN*, *flgB*, *flgD*, *flgE*, *flgF*, *flgG*, *flgH* and *flgI*) while the genes clustered in this same functional class that are up-regulated in IST439 relate with chemotaxis (*flhD*, *motA*, *motB*, *cheY*, *tar*, *BCAL0136*). A detailed description of selected functional groups of genes differently expressed in IST439 and IST4113 follows.

### Genes involved in translation are up-regulated in the clonal variant more resistant to antibiotics targeting this cellular process

**Figure 2:**
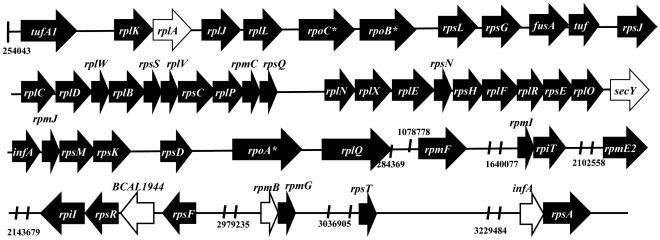
Genetic organization of genes related with translation (ribosomal genes and translation initiation factors) found to be up-regulated in the IST4113 variant, compared to IST439. Black arrows indicate the genes with higher transcript levels in IST4113 than in IST439 whereas white arrows indicate those equally transcribed in the two variants. The localization of the genes in the genome is provided using the genome of the sequenced J2315 as a reference, according with the information available in the *Burkholderia* Genome Database. Genes marked with an asterisk are not related with translation.

The clonal variant IST4113 is significantly more resistant than IST439 to the aminoglycosides tobramycin (MIC of 512 µg mL^−1^, compared to 128 µg mL^−1^) and gentamicin (MIC of 1400 µg mL^−1^, compared to 130 µg mL^−1^) [15], [16] that target protein synthesis. Eighty-six genes related with translation were found to be more actively transcribed in IST4113, compared to IST439, including components of the large and small ribosome subunits and initiation and elongation factors (Supplementary Table S1). These genes are distributed throughout the several rRNA operons present in chromosome 1 (Fig. 2). The levels of a significant number of tRNAs were also found to be higher in IST4113 (Supplementary Table S2) reinforcing the idea that translation is a more active process in this clonal variant.

### *B. cenocepacia* genes involved in antibiotic resistance are differently expressed in IST439 and IST4113

**Figure 3:**
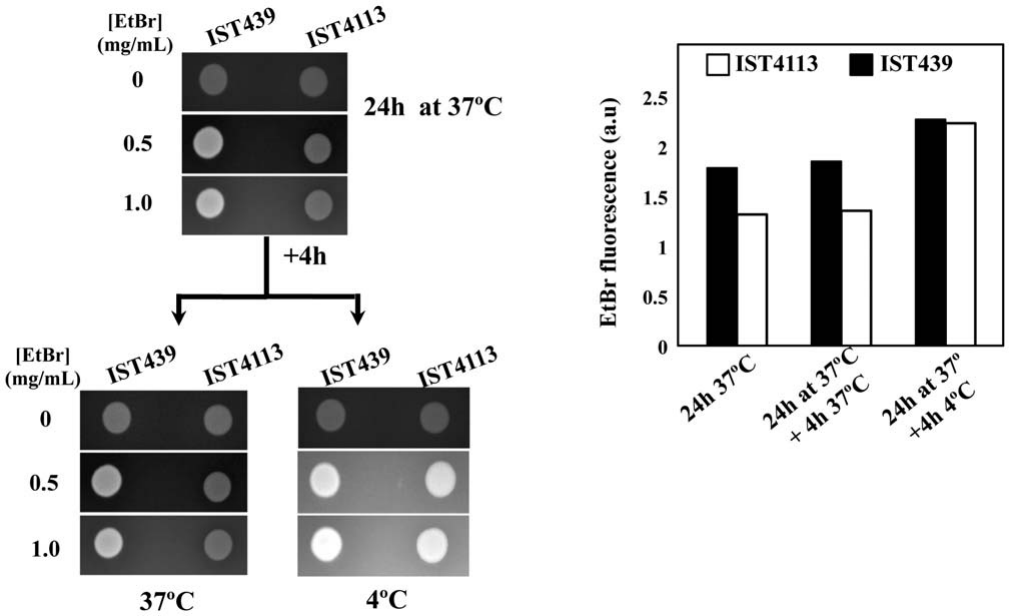
Indirect comparison of the active drug export capacity of IST439 and IST4113, based on the ethidium bromide screening agar method. Cells of the two clonal variants were harvested in mid-exponential phase of growth and spread, in duplicate, onto the surface of LB plates supplemented or not with ethidium bromide, as detailed in Materials and Methods. The plates were incubated for 24 h at 37°C and the fluorescence values emitted by the cell mass present in each plate were quantified. One set of plates was left to grow for an additional period of 4 h at 37°C while the other set was transferred to 4°C, a temperature at which the activity of MDR pumps is significantly reduced. The results obtained are representative of at least three independent experiments.

Besides being more resistant to aminoglycosides, the clonal variant IST4113 is also more resistant than IST439 to all the antimicrobials of the different classes tested, including the β-lactams piperacillin, tazobactam, ceftazidime and imipenem; the fluoroquinolone ciprofloxacin and the folate-pathway inhibitors trimethoprim-sulfamethoxazole [15], [16]. This increased antibiotic-resistance phenotype of IST4113 prompted us to search for genes previously related with antimicrobial resistance within the microarray dataset (Table 2). Among the potential drug resistance determinants identified before in the genome of *B. cenocepacia* J2315 [22], nine are differently expressed in IST439 and IST4113 (Table 2). Those up-regulated in IST4113 include *mdtABC* and *bpeA*, whose products are two pumps of the resistance nodulation-division (RND) family; *BCAM0201*, a drug pump of the Major Facilitator Superfamily (MFS); and *BCAM2188*, encoding a component of an ABC-transporter thought to be involved in the extrusion of macrolides (Table 2). Remarkably, the elimination of *bpeA* was proved to increase *B. cenocepacia* susceptibility to gentamicin, tobramycin and ciprofloxacin [27]. The transcript levels of *BCAM0791*, predicted to encode a multidrug MFS efflux pump, were also found to be higher in IST4113 than in IST439 (Table 2). The higher transcription level of a number of these drug pump-encoding genes in IST4113 variant was confirmed by qRT-PCR (Table 2). Although the transcript levels of most of these genes vary in the two variants from 1.5 to 2.3-fold only, minor changes in the expression of bacterial drug pumps have impact in drug resistance [28], [29], [30]. The quantification of the intracellular accumulation of the fluorescent dye ethidium bromide in the two clonal variants reinforce the idea that IST4113 is able to extrude drugs more actively than IST439, given that IST4113 cells accumulate a lower amount of ethidium bromide than IST439 cells after 24 h of cultivation at 37°C in LB plates (Fig. 3). The transference of both culture plates to 4°C, a temperature known to drastically inhibit the activity of MDR pumps, led to a similar accumulation of the dye in the two isolates (Fig. 3), consistent with the proposed higher drug active export capacity of IST4113 cells.

**Table 2:** Antibiotic resistance-genes differently transcribed in IST439 and IST4113.

CDS/Gene	Function	mRNA IST4113/mRNA IST439	
		Microarray analysis	qRT-PCR
*Chromossome 1*			
BCAL1079/*mdtA*	Multidrug efflux transporter of the Resistance Nodulation Family (known as RND6)	1.6	1.5
BCAL1079/*mdtB*		1.6	2.2
BCAL1079/*mdtC*		1.7	7.0
BCAL2822/*bpeA*	Subunit of the drug efflux transporter RND4	1.6	-
BCAL1076/*ompA*	Outer membrane protein	−1.7	-
BCAL1510	Outer membrane protein	−2.3	-
BCAL1511/*emrA*	Putative multidrug resistance transporter	−1.7	-
BCAL1829	Outer membrane protein	−5.3	-
BCAL2205	D-alanyl-D-alanine endopeptidase (penicillin-binding protein precursor)	−1.6	-
BCAL2645	Outer membrane protein; has some similarity with *P. aeruginosa* OprF	−2.3	-
BCAL2958/*ompA*	Outer membrane protein	−1.8	-
BCAL3008	Outer membrane protein; has some similarity with *P. aeruginosa* OprE	−5.2	-
BCAL3110/*waaA*	Protein involved in incorporation of 3-deoxy-D-manno-octulosonic-acid in lipid A during LPS biosynthesis	−1.6	-
BCAL3473	Outer membrane protein	−1.6	-
*Chromossome 2*			
BCAM0201	MFS efflux transporter	1.6	1.7
BCAM2188	Component of a putative macrolide-specific efflux transporter	2.2	-
BCAM0791	MFS efflux transporter	2.3	2.2
BCAM1015	Outer membrane protein	−2.7	-
BCAM1738	Putative penicillin-binding protein	−1.8	-
BCAM1787	Outer membrane protein	−1.7	-
BCAM1931	Outer membrane protein; homologous to *B. cepacia* Opcp1	−3.4	-
BCAM2385/*arr*	Rifampin ADP-ribosyl transferase	−1.7	-
*Chromossome 3*			
BCAS0016	Transporter of the fusaric acid resistance family	−2.0	-
BCAS0460	Outer membrane protein	−2.1	−7.5

The microarray dataset of genes differently transcribed in IST439 and IST4113 was searched for antibiotic-resistance determinants identified by Holden *et al.*, 2009 and Bazzini *et al.*, 2011. To confirm the results obtained in the microarray analysis, the transcript levels from some of the genes listed were compared by quantitative real time RT-PCR (qRT-PCR).

Multiple antibiotic resistance in Bcc bacteria has also been related with the expression of antibiotic modifying enzymes and reduced permeability of the outer membrane [1], [2], [31], [32]. However, the transcript levels from the four β-lactamases encoded by J2315 genome (*BCAM0393*, *BCAM1779*, *BCAM2165* and *BCAS0156*) are identical in the two variants and no alteration was registered in the transcript levels from genes encoding enzymes required for inactivation of aminoglycosides (the aminoglycoside-3-phosphotransferase *BCAM0928* and the aminoglycoside-3′adenyltransferase *BCAM1013A*). The transcription of a penicillin-binding protein (PBP), *BCAM1738*, and of a PBP-precursor, *BCAL2205*, was down-regulated (by 1.8 and 1.6-fold, respectively) in IST4113 (Table 2). Since ceftazidime and piperacillin directly target PBP activity, the lower expression of these two genes is likely to favour IST4113 resistance to these antibiotics.

The reduced permeability of *B. cenocepacia* outer membrane registered in cells adapted or intrinsically resistant to antibiotic stress has been mainly related with the down-regulation of porins and with a reduced content of 3-deoxy-D-manno-octulosonic-acid and phosphate in the lipopolysaccharide (LPS), a modification of the cell surface that leads to the decrease of binding sites for cationic antibiotics thus preventing their diffusion to the intracellular environment [1], [31], [32]. Remarkably, the transcript level of the *waaA* gene, whose product catalyses the transference of 3-deoxy-D-manno-octulosonic-acid into lipid A, is reduced in IST4113 (Table 2) suggesting the presence of a more positive LPS in IST4113 cells compared to IST439 cells. This hypothesis is also consistent with the higher resistance levels of IST4113 to cationic aminoglycosides [16]. Ten genes encoding outer membrane porins (*ompA*, *BCAL1076*, *BCAL1829*, *BCAL2645*, *BCAL3008*, *BCAL3473*, *BCAM1787*, *BCAM1931*, *BCAM1015* and *BCAS0460*) were found to be down-regulated in IST4113, compared to IST439, and the lower transcript levels of some of these genes in IST4113 cells was confirmed by qRT-PCR (Table 2). Notably, *BCAM1931* is highly homologous (89% identity) to the *B. cepacia* outer membrane porin Opcp1, through which β-lactams can diffuse into the cytosol [32] and *BCAL3008* and *BCAM1931* are similar (30% identity) to the *E. coli* porins OmpF and OmpC, respectively, that also serve as channels for the entry of β-lactams [33]. None of the other outer membrane porins whose expression is suggested to be reduced in IST4113 was previously associated with antibiotic resistance in *B. cenocepacia*.

### Metabolic adaptation of *B. cenocepacia* during long-term colonization of a CF patient

Approximately 20% of the genes differently expressed in the clonal variants herein examined are related with cellular metabolic activity: 101 genes are involved in the metabolism of carbohydrates, 54 in the metabolism of amino acids and other nitrogen compounds, 22 in lipid metabolism, 13 in the metabolism of sulphur compounds and 10 genes are related with nucleotide metabolism (Fig. 1, Supplementary Table S1 and Supplementary Tables S2 and S3). Such a high number of genes related with metabolic functions being differently transcribed in IST439 and IST4113 indicates that an extensive metabolic adaptation has occurred during the colonization period that separates the dates of isolation of these two variants. The most relevant adaptive responses suggested by this transcriptomic analysis are detailed below:

#### *i) Central carbon metabolism and energy production*

**Figure 4:**
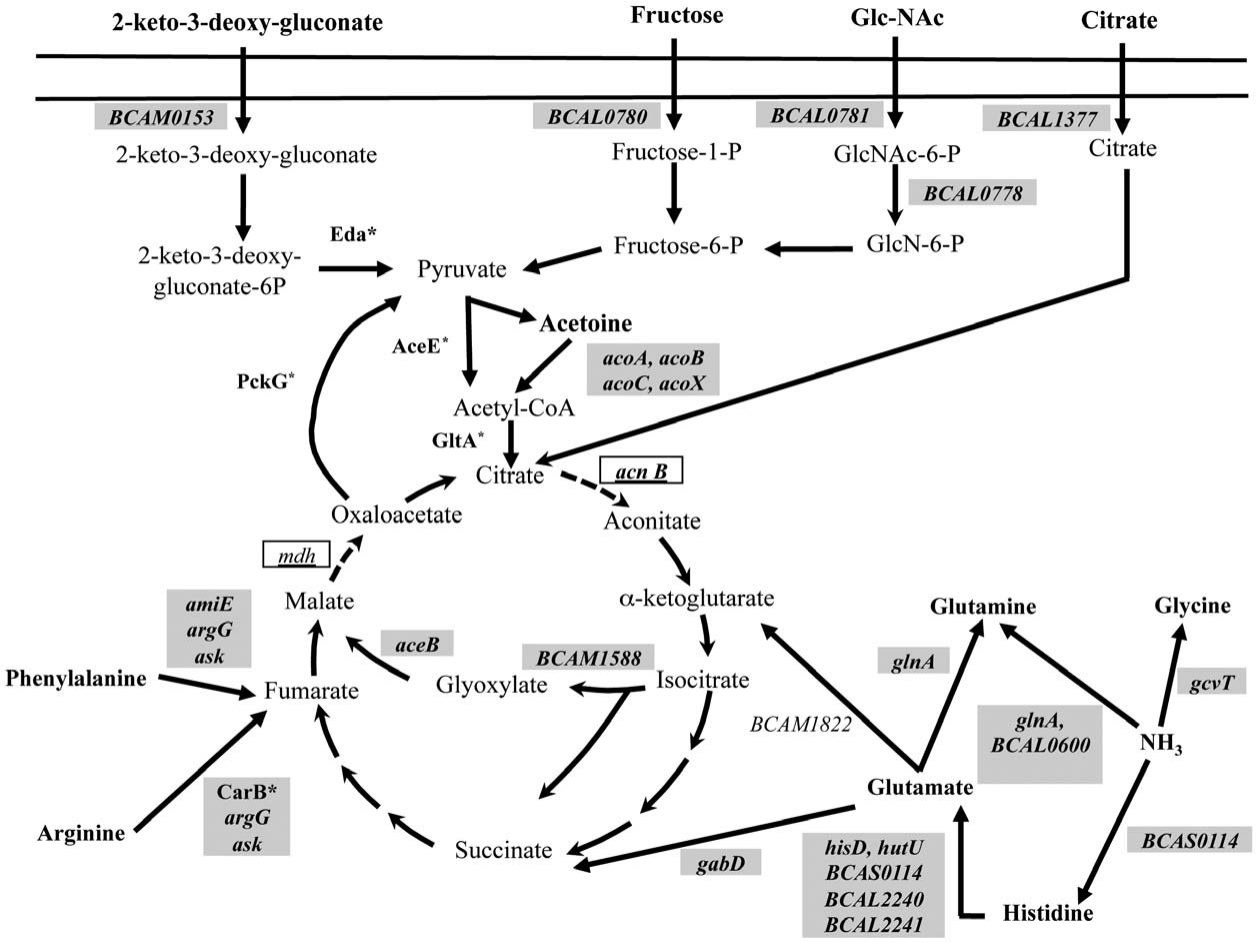
Genes differently transcribed in IST439 and IST4113 related with the uptake and metabolism of carbohydrates and amino acids, shown associated to the metabolic pathways in which they are involved. The metabolic map shown was prepared based on the information available in the KEGG Pathways Database and using as the input dataset the genes clustered in the functional classes shown in Fig. 1 “Transport”, “Amino acid metabolism” and “Carbohydrate Metabolism”. The genes that are more actively transcribed in IST4113 are shown inside grey boxes whereas the genes that have a higher transcript level in the IST439 variant are indicated inside white boxes. Proteins known to be more abundant in IST4113 than in IST439, based on the results of a previous expression proteomic analysis [16], are marked with an asterisk.

The genes related with central carbon metabolism that have an altered expression in the clonal variants IST439 and IST4113 were clustered according to the metabolic pathways in which they are involved, using the KEGG pathways database (Fig. 4). Genes encoding enzymes involved in the catabolism of citrate, fructose, acetoin, N-acetylglucosamine and 2-keto-3-deoxygluconate and genes encoding transporters required for the uptake of these same sugars were found to be up-regulated in IST4113 (Fig. 4, Supplementary Table S1 and Supplementary Table S2). Two enzymes of the glyoxylate cycle, isocitrate lyase (*BCAM1588*) and malate synthase (*aceB*), are also more actively transcribed in IST4113 cells (Fig. 4 and Supplementary Table S1) suggesting an increased flux of this anaplerotic pathway in this clonal variant, compared to IST439.

The genes down-regulated in IST4113 that were clustered in “carbohydrate metabolism” functional class include those involved in phenylacetate catabolism (*paaD*, *paaC*, *paaK*, *paaI*, *paaG*, *paaF*, *paaZ* and *BCAM2568*), Krebs cycle (*BCAL2746*, *BCAM0964*, *acnB* and mdh) and electron transport (Supplementary Table S1, Supplementary Table S3). In particular, seven genes encoding subunits of two cytochromes with a low affinity to oxygen (*cyoABCD*, encoding subunits of cytochrome *o* and *petABC* whose products are three subunits of ubiquinol-cytochrome-*c* reductase) were found to be repressed in IST4113 (Supplementary Table S1 and Supplementary Table S3). In contrast, *BCAM2674* and *BCAM2675* genes, which encode two homologues of *P. aeruginosa* cytochrome CioAB, demonstrated to have a high affinity for oxygen and to serve as electron donor to this gas under microaerophilic conditions [34], were found to be up-regulated in IST4113 cells by 2.7- and 1.6-fold, respectively (Supplementary Table S1).

#### *ii) Amino acid and nitrogen metabolism*

The transcript levels of 46 genes encoding ABC transporters involved in the uptake of amino acids and oligopeptides, as well as of genes involved in the metabolism of amino acids into sugar precursors, are higher in IST4113 than in IST439 (Fig. 4, Supplementary Table S1 and Supplementary Table S2). Since it is known that amino acids are present at high concentrations in the sputum of CF patients, especially after periods of exacerbated bacterial infection [35], [36], this transcriptional response may reflect the long-term adaptation of *B. cenocepacia* to the nutritional environment of the CF lung. The up-regulation of genes related with the uptake of amino acids was also observed when *B. cenocepacia* was cultivated in the presence of CF sputum [24]. Furthermore, three genes involved in the reduction of ammonium into amino acids were also found to be up-regulated in IST4113: *glnA*, encoding glutamine synthethase; *BCAS0014*, encoding a predicted histidine ammonia-lyase and *gcvT*, encoding a component of the glycine aminomethyltransferase system (Fig. 4).

#### *iii) sulphur metabolism*

**Figure 5:**
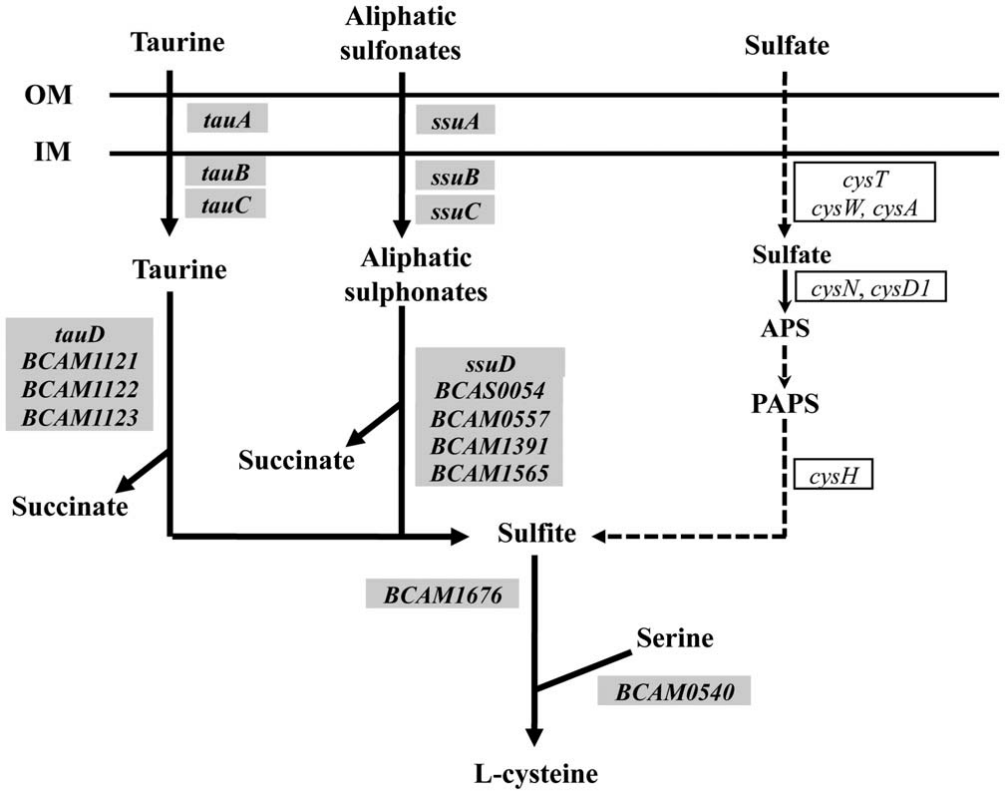
Genes related with sulphur metabolism differently transcribed in IST439 and IST4113 cells. The metabolic map was prepared based on the information available in the KEGG Pathways Database using as the input dataset the genes clustered in the functional classes shown in Fig. 1 “Transport” and “Sulphur metabolism”. The genes more actively transcribed in IST4113 are indicated inside grey boxes whereas the genes that have a higher expression in IST439 are indicated inside white boxes.

The transcription of the three genes of the *tauABC* operon, which encodes the ABC-transporter for the sulphur-containing amino acid taurine, whose concentration is high in CF patients' sputum [37], and of four genes encoding taurine dioxygenases (*tauD*, *BCAM1121*, *BCAM1122*, *BCAM1123*), required for the reduction of taurine into sulfite, was found to be up-regulated in IST4113 (Supplementary Table S1; Fig. 5). Genes involved in the uptake and metabolization of monoalkanesulphonates are also more actively transcribed in IST4113 (Fig. 4) while the genes of the sulphate metabolism cluster (*cysN*, *cysD1*, *cysH*, *sbp*, *cysT* and *cysW*) have reduced transcript levels in these same cells, compared to IST439 (Supplementary Table S1 and Fig. 5). Interestingly, many natural sulphonates are known to be present in the surface of epithelial lung cells and in mucin [38] and clinical isolates of *B. cenocepacia* were found to have a high mucin sulphatase activity [39].

### Genes involved in iron uptake are up-regulated in the IST4113 isolate

The transcription of seven out of the twenty genes that compose the two operons of the ornibactin biosynthetic cluster were found to be up-regulated in IST4113, compared to IST439 (Supplementary Table S1). Although the *orbI* gene belongs to the same transcription unit as *orbJ* and *orbK* [40], only these two last genes were identified in our dataset as being transcriptionally activated in the IST4113 variant (Supplementary Table S1). However, qRT-PCR experiments showed that the transcript levels of *orbI* gene are also higher in IST4113 cells (results not shown). Notably, results from previous studies suggest that ornibactin biosynthesis is essential in *B. cenocepacia* colonization of the lungs of a CF rat model [41]. Other genes involved in iron uptake were also found to be transcriptionally activated in IST4113 including *BCAL1371*, *BCAM0564* and *BCAM1571*, which encode three TonB-dependent receptors of iron-siderophores, and *huvA* and *hmuS*, which encode two of the four genes of a cluster dedicated to the uptake of heme or hemin.

### *B. cenocepacia* virulence genes and other relevant genes differently expressed in the two variants

The transcription levels from several genes thought to be virulence determinants in *B. cenocepacia* are different in IST439 and IST4113 (Table 3). The virulence-related genes up-regulated in IST4113 include two adhesins (*BCAM0219* and *BCAM0223*), a flavohemoprotein (*hmpA*), the enzymes of the ornibactin biosynthetic pathway and the quorum sensing-regulated transcription factors *cciR* and *shvR*. On the other hand, twenty-six known virulence genes have a reduced transcription level in the IST4113 variant including the exoproteases ZmpA and ZmpB, two secreted phospholipases (*plcN* and *BCAM0408*), several components of type III and type VI secretion systems, two enzymes involved in LPS biosynthesis (*waaA* and *wbxY*), the ^54^σ factor RpoN and the cable pili adhesin *adhA* (Table 3).

**Table 3:** *B. cenocepacia* virulence determinants differently expressed in IST439 and IST4113.

CDS/Gene	Function	mRNA IST4113
		mRNA IST439
*Chromossome 1*		
BCAL1697/*orbJ*	Ornibactin biosynthesis non-ribosomal peptide synthase	1.9
BCAL1698/*orbK*	Ornibactin biosynthesis protein	3.0
BCAL1699/*pvdA*	L-ornithine -oxygenase required for iron assimilation	4.9
BCAL1700/*orbA*	Ornibactin receptor	5.9
BCAL1701/*pvdF*	Ornibactin synthetase F	4.1
BCAL1702/*orbL*	Putative ornibactin biosynthesis protein	3.1
BCAL3285/*hmpA*	Flavohemoprotein associated to phenylacetate catabolism	1.5
BCAL0212/*paaD*	Phenylacetic acid degradation protein PaaD	−1.7
BCAL0341	Putative type VI secretion system protein TssB	−1.6
BCAL0344	Putative type VI secretion system protein TssE	−1.5
BCAL0813/*rpoN*	Putative RNA polymerase sigma-54 factor	−1.5
BCAL1046/*plcN*	Putative non-hemolytic phospholipase C	−1.7
BCAL3110/*waaA*	3-deoxy-D-manno-octulosonic-acid transferase involved in biosynthesis of O-antigen	−1.6
BCAL3111/*wbxY*	Protein involved in biosynthesis of O-antigen	−1.6
BCAL3299/*katB*	Peroxidase/catalase KatB	−1.6
*Chromossome 2*		
BCAM0207	Kinase associated to exopolysaccharide biosynthesis	1.6
BCAM0214	Glycosyltransferase associated to LPS biosynthesis	2.9
BCAM0219	Putative haemagglutinin-related autotransporter protein	1.5
BCAM0223	Putative haemagglutinin-related autotransporter protein	3.8
BCAM0240/*cciR*	N-acylhomoserine lactone dependent regulatory protein	1.7
BCAS0296	Other potential surface polysaccharides	1.6
BCAM0338	Protein of unknown function	−1.6
BCAM0339	Hypothetical protein	−1.6
BCAM0408	Putative phospholipase C	−2.2
BCAM2048/*bcscN*	Type III secretion system protein	−1.7
BCAM2050/*bcscK*	Type III secretion system protein	−1.7
BCAM2051/*bcscJ*	Type III secretion system protein	−1.7
BCAM2052	Protein of unknown function of the type III secretion system	−1.7
BCAM2053	Protein of unknown function of the type III secretion system	−2.8
BCAM2055/*bcscC*	Type III secretion system protein	−1.8
BCAM2056/*bcscS*	Type III secretion system protein	−1.8
BCAM2143/*adhA*	Cable pilus associated adhesin protein	−2.0
BCAM2169	Putative outer membrane autotransporter	−2.4
BCAM2307/*zmpB*	Zinc metalloprotease	−3.5
*Chromossome 3*		
BCAS0225/*shvR*	Shiny variant regulator	1.5
BCAS0296	Other potential surface polysaccharides	1.6
BCAS0305	Putative lipoprotein	2.2
BCAS0236	Putative haemagglutinin-related autotransporter protein	−2.3
BCAS0293/*aidA*	Nematocidal protease AidA	−9.1
BCAS0409/*zmpA*	Zinc metalloprotease ZmpA	−7.8

The dataset of genes differently transcribed in IST439 and IST4113 was searched for genes known to be involved in virulence using the compilation made by Holden *et al*., 2009 and Loutet and Valvano, 2010 [78]. To confirm the results from the microarray analysis, the transcript levels from a number of genes listed was compared by quantitative real time RT- PCR (qRT-PCR).

Beside the two above referred adhesins, the transcript levels from a number of other genes related with adhesion were also higher in the IST4113 variant, including the lectins *bclB* and *bclC* that mediate bacterial attachment to mucin [42], [43], [44] and several genes related with the assembly of flagella (*flhB*, *flhF*, *fliG*, *fliK*, *fliJ*, *fliN*, *flgB*, *flgD*, *flgE*, *flgF*, *flgG*, *flgH*, *flgI*) which was found to play an essential role in the initial steps of adhesion to epithelial cells [45]. In contrast, the transcript level of the major cable-pili adhesin *adhA* [46] is lower in IST4113 than in IST439 (Supplementary Table S1). Two genes of the O-antigen biosynthesis cluster, *waaA* and *wbxY*, are down-regulated in IST4113, suggesting a decreased O-antigen content in the LPS of these cells, compared to IST439 (Supplementary Table S1), consistent with the rough morphology of IST4113 colonies and the smooth morphology of IST439 colonies [15], [47]. Other relevant genes up-regulated in the IST4113 variant include the GTP-binding exoprotease *lepA*, two zinc metallo-endopeptidases, *BCAL1671* and *BCAM0180*, the periplasmic serine protease inhibitor ecotin (encoded by the *eco* gene) and *BCAM0550*, a putative secreted lipase (Supplementary Table S1). Interestingly, *P. aeruginosa* ecotin was suggested to play a role in improving persistence of this bacterium in the CF lung by protecting this bacterium against the activity of neutrophils elastase [48].

## Discussion

During chronic infections the airways of a patient with CF represent an evolving system with multiple phenotypic variants emerging from the clonal population and becoming established in the patient's airways [6], [7], [9], [15], [49]. These clonal variants may result from genetic adaptation and selection pressures exerted by the host environment, in particular, from the challenges imposed by the immune defences, antimicrobial therapy, nutrient availability and oxygen limitation. Results from the transcriptomic analysis described in this study provide important indications on the adaptive strategies used by *Burkholderia cenocepacia*. The large number of genes (above 1000) found to be differently expressed in the two *B. cenocepacia* isolates examined, recovered in the beginning of the infection (IST439) and after three years of chronic colonization and lung deterioration (IST4113), reflects the marked alteration of the genomic expression that occurred in these clonal variants, as described before for *P. aeruginosa* [6], [7], [9], [12].

The two variants examined belong to the same clonal complex as *B. cenocepacia* J2315 [15] and harbour in their genome DNA regions specifically found in this strain and in other members of the epidemic ET-12 lineage, namely, the pathogenicity island *cci*. One of the genes located in this pathogenicity island that is up-regulated in IST4113 is the quorum-sensing (QS) regulator CciR which plays a role in the persistence of *B. cenocepacia* infections [23], suggesting that the transcription level of this gene and of two other genes located in the pathogenicity *cci* island (*BCAM0238* and *BCAM0243*) may be enhanced during long-term colonization and the progress of the disease.

An impressive number of genes related with bacterial adaptation to the nutritional environment of the CF lung emerged from this study as having an altered transcription during long-term colonization. It is known that bacterial human pathogens frequently adjust their metabolic pathways to sustain growth in a hostile host-specific microenvironment [50]. The transcript levels of genes encoding amino acid transporters or involved in amino acid catabolism were higher in isolate IST4113, in line with previous reports for *P. aeruginosa* [6], [10], [35], [51], [52], [53]. During chronic respiratory infections, *B. cenocepacia* cells grow and proliferate within the viscous layers of the sputum present in the patients' lungs, a microenvironment with a complex nutrient composition that includes a high concentration of amino acids [35], [52], [53]. The transcript levels of three genes involved in the reduction of ammonium into amino acids (*glnA*, *gcvT* and *BCAS0014*) are also higher in the IST4113 variant, compared to IST439, suggesting that *B. cenocepacia* may use a nitrogen-carbon balancing mechanism dependent on ammonium reduction, similar to the one reported for *P. aeruginosa* [54]. An increased activity of the glyoxylate cycle in IST4113 is also hypothesized, based on the higher transcript levels of isocitrate lyase- and malate synthase- encoding genes registered in these cells, compared to IST439. Moreover, the transcript levels of the RpoN σ factor, known to repress the glyoxylate pathway in *P. aeruginosa* [55], were lower in IST4113 than in IST439. A higher activity of glyoxylate cycle at later stages of the infection may allow the bacterium to use available C2 substrates as the sole carbon sources, in particular, the fatty acids present in surfactants existing in the CF lung [1]. Indeed, four genes encoding enzymes related with fatty acid β-oxidation (*phbA*, *BCAM2526*, *BCAS0346* and *BCAS0419*) are more actively transcribed in IST4113 than in IST439 (Supplementary Table S1). Interestingly, isocitrate lyase is considered an important virulence factor in human-pathogenic bacteria because it allows survival in the nutrient-depleted environment of the phagolysosome [56], [57]. Although *B. cenocepacia* isolates retrieved from chronically infected CF patients have been demonstrated to grow inside macrophages [58], the role of isocitrate lyase and of the glyoxylate cycle in this phenomenon has never been addressed.

A number of genes related with iron uptake were also found to be up-regulated in IST4113 which exhibits a higher growth efficiency under iron limitation [15]. These up-regulated genes included genes required for the biosynthesis of the iron siderophore ornibactin and several TonB-dependent receptors that are required for the uptake of iron-siderophore complexes. Iron-acquisition mechanisms are considered key factors for the successful colonization of human infecting bacteria as the level of free iron within mammals is well below the one required for optimal bacterial growth [59]. In particular, ornibactin biosynthesis is essential for long-term *B. cenocepacia* infections of rat lungs [41]. The use of the heme group released upon proteolytic degradation of heme-containing proteins (e.g. lactoferrin, ferritin and hemoglobin) is also considered a crucial mechanism by which human infecting bacteria scavenge iron [59] and *B. cenocepacia* is able to use ferritin as an iron source [60]. Significantly, the *huvA* and *hmuS* genes, involved in the uptake and degradation of heme, are up-regulated in the IST4113 variant, compared to IST439. Interestingly, one of the exoproteases found to be up-regulated in the IST4113 variant, LepA, was recently demonstrated to be involved in the ability of *P. aeruginosa* to use hemoglobin as a carbon and iron source [61].

Microaerobic respiration was recently demonstrated to be the predominant mode of *P. aeruginosa* growth in the CF lung [62] and proposed to occur during *B. cenocepacia* chronic CF infections [15]. The increased transcript levels from genes encoding CioAB cytochrome subunits, which are known to be used for electron transference to oxygen under microaerophilic conditions [62], and the lower transcript levels from genes encoding cytochromes used for electron transfer under aerobic conditions (cytochromes *o* and *bd* subunits) registered in the IST4113 variant, reinforces the concept that microaerobic respiration occurs and *B. cenocepacia* adapts to growth in the airways of CF patients during long-term colonization and the progression of the disease accompanied by deterioration of pulmonary function.

A number of genes considered as *B. cenocepacia* virulence factors do have an altered transcription in the two variants (Table 3). In particular, the haemagglutinin autotransporters *BCAM0219* and *BCAM0223*, proposed to be involved in *B. cenocepacia* adhesion to the epithelial lung tissue [63], are up-regulated in IST4113. *BCAM0219* and *BCAM0223* encode two orthologues of the surface-attached collagen-binding protein YadA from *Yersinia* species that, together with *BCAM0224*, form an adhesin-cluster considered an extension of the *cci* pathogenicity island [63]. Notably, the expression of *BCAM0223* increases *B. cenocepacia* survival in the lungs of a CF rat model [64]. Other responses favouring the adhesion of *B. cenocepacia* to epithelial cells are also suggested to be improved in IST4113 cells, based on the results of the microarray analysis, including the increased expression of genes involved in biosynthesis and assembly of the flagella and the production of a smaller O-antigen chain in the LPS [45], [65]. *B. cenocepacia* attaches to the mucin present in the airways of CF patients through the expression of lectins that recognize protruding glycans [42], [43], [44]. Four lectin-encoding genes were found to be differently transcribed in IST439 and IST4113: *bclB* and *bclC* are up-regulated in IST4113, while *bclA* and *BCAS0292* are down-regulated. BclC is involved in the recognition of fucosylated glycans while BclA is involved in the recognition of mannosylated glycans [42], [43]. Compared to the mucin of healthy individuals, the mucin recovered from CF patients has a much higher content of fucosylated sugars and almost no mannose [66], consistent with the adaptive transcriptional response registered for IST4113 cells. The virulence factors suggested to be expressed at lower levels in IST4113 include the exoproteases ZmpA and ZmpB, the cable pili adhesin AdhA, several proteins of type III and type VI-secretion systems and the phospholipases *plcN* and *BCAM0408* (Table 3). Since these proteins can be recognized by the host immune system, either because they are secreted or because they are present at the cell surface, their decreased amount may help *B. cenocepacia* to circumvent the activity of the host immune system, as proposed for *P. aeruginosa* [6], [8], [67].

The IST4113 clonal variant is more resistant than the first isolate to a wide variety of clinically relevant antibiotics with very different biological targets [15], [16]. Based on the results of this study, the mechanisms underlying this enhanced antibiotic resistance of IST4113 cells involve a higher active drug export capacity related with the increased transcription from various genes encoding drug efflux pumps in IST4113 (Table 2). Among these drug pump-encoding genes up-regulated in IST4113 are three subunits of the RND-efflux transporter *mdtABC* (known as RND6) and one component (*bpeA*) of the RND efflux pump *BCAL2821*-*BCAL2820*-*bpeA* pump (known as RND4). The transcript levels of *BCAL2821* and *BCAL2820* genes that do not belong to the same transcriptional unit of *bpeA* [30], were identical in the two variants. RND4 efflux pump is involved in the extrusion of gentamicin, tobramycin and ciprofloxacin in *B. cenocepacia* J2315 [27], [29], [68], three antibiotics to which IST4113 is less susceptible than IST439 [15], [16]. No substrates had been identified for the MdtABC pump or for the other putative drug efflux pumps found to be up-regulated in IST4113. The RND4 pump is highly homologous to the *P. aeruginosa* drug pump MexXY-OprM, demonstrated to have a role in the intrinsic and acquired resistance of CF isolates of *P. aeruginosa* to aminoglycosides [69], [70]. The up-regulation of MexXY-OprM in response to aminoglycosides and other protein synthesis inhibitors was related with ribosomal activity, establishing a link between the efflux of these drugs and translational machinery activity [71]. Interestingly, more than eighty genes related with translation were found among the set of genes up-regulated the IST4113 variant.

The alteration of the bacterial cell surface is another mechanism contributing to the intrinsic and acquired resistance of Bcc bacteria to antibiotics [2], [31], [32]. In particular, the down-regulation of porine synthesis in CF isolates of *B. cenocepacia* resistant to antibiotics was described [32]. Ten genes encoding porines were found to be down-regulated in IST4113, including a few genes encoding proteins highly homologous to porines demonstrated to serve as channels for the entry of antibiotics in other Gram negative bacteria. Several lipoproteins and proteins related with LPS and peptidoglycan biosynthesis were also differently expressed in IST439 and IST4113. These observations together with the different morphology of the colonies formed by the two variants (IST439 colonies are smooth and IST4113 colonies are rough [16]) strongly suggest that they have a different cell surface, a phenotypic trait that can contribute to the higher resistance of IST4113 to antibiotics since the conversion from smooth to rough LPS in *P. aeruginosa* results in a higher cell impermeability to aminoglycosides [72].

Several transcriptional regulators were also found to be transcribed at different levels in IST439 and IST4113. Among these are CciR and ShvR, two transcription factors with a role in quorum-sensing-mediated signalling and in the control of *B. cenocepacia* genomic expression and pathogenesis [23], [73], [74], [75]. Remarkably, 37 genes described as being activated by CciR and 57 genes documented to be repressed by this transcription factor are present in the dataset of genes differently expressed in IST4113, compared to IST439 (Supplementary Table S4). Although ShvR plays a crucial role in the control of *B. cenocepacia* genomic expression [75], there is no information on its target genes. The dataset of genes differently transcribed in IST439 and IST4113 also include a number of known targets of other transcription factors (e.g. CepR) whose transcript levels were not found to be significantly different in the two variants. All these transcription regulators deserve further in depth studies to elucidate their eventual role in genomic expression reprogramming during long-term colonization under antibiotic therapy and the progression of the disease.

The number of genes found to produce different mRNA levels in IST439 and IST4113 was much higher than the number of proteins previously found to be differently abundant in these two isolates (1024 compared to 79, using the same 1.5-threshold level and cells grown under identical conditions [16]). The limited protein coverage of the 2-DE-based quantitative proteomic analysis carried out failed to identify a number of relevant gene expression differences that emerged from the present study but, in general, the functional classes enriched in the datasets of differently abundant proteins or transcripts coincided (Supplementary Table S5). In particular, the up-regulation of genes/proteins involved in translation, iron binding and uptake and in the biogenesis of cell envelope and outer membrane in the IST4113 variant, compared to IST439, was identified in the two genome-wide expression analyses (Supplementary Tables S1, S2, S3 and S5). However, the present study reveals a number of genes that were ignored in the proteomic analysis and extends the knowledge to genes of functional groups whose identification is not possible through the quantitative proteomic approach used, as it is the case of transmembrane solute transporters, in particular drug-pumps, and to low expressed genes, namely those encoding transcription regulators. The present transcriptomic analysis together with the extensive phenotypic studies of serial isolates of *B. cenocepacia* obtained during long-term colonization of the same CF patient [15], [16], [76], is providing clues that strongly suggest a genetic adaptation to the challenges exerted by the immune system, antimicrobial therapy, nutrient availability and microaerophilic conditions, leading to a higher antibiotic resistance and bacterial persistence in the lungs. Understanding these mechanisms is crucial for an improved therapeutic outcome of chronic infections in CF patients.

## Materials and Methods

### Bacterial isolates and growth media

The two *Burkholderia cenocepacia* clinical isolates examined in this study, IST439 and IST4113, were recovered from the sputum of an anonymous CF patient, under surveillance at the CF Center of Hospital of Santa Maria on January 1999 and on November 2001, respectively [76]. The studies performed by our laboratory involving the use of *B. cenocepacia* isolates collected from CF patients followed at Hospital de Santa Maria performed have been approved by the ethics committee of the Hospital. IST439 and IST4113 isolates are two clonal variants characterized before [15], [16]. Bacterial growth was carried out in Luria Bertani (LB) medium (Difco), at 37°C, with orbital agitation (250 rpm) or on solid LB medium obtained by supplementation of the liquid LB medium with 2% agar (Iberagar, Portugal).

### Genome-wide transcriptomic analysis of *B. cenocepacia* IST439 and IST4113 isolates

#### a) Growth conditions and RNA extraction

*B. cenocepacia* IST439 and IST4113 were cultivated in LB liquid growth medium until mid-exponential phase (OD_640 nm_ 0.4±0.05) and then diluted to a standardized OD_640 nm_ of 0.2±0.05 in NaCl 0.9% (w/v). These cell suspensions (100 µl) were plated onto LB agar plates and then incubated for 24 h at 37°C. To reduce biological variation, cells harvested from 8 independently prepared LB-agar plates were mixed together and used for subsequent extraction of total RNA. RNA extraction was made using the RiboPure kit (Ambion) according with the manufacturer's instructions. The recovered RNA was treated for one hour with 10 U of DNaseI, precipitated with 7.5 M lithium chloride and finally resuspended in 100 µL of RNase-free water. The samples were concentrated to a minimum of 1.25 µg/µL and the quality of the RNA was assessed upon electrophoretic separation on a Bioanalyzer. Only samples with RNA integrity numbers higher than 8.0 were used for the subsequent microarray analysis.

#### b) Microarray hybridization and data analysis

The custom-made 4×44 K microarrays for *B. cenocepacia* as well as the protocols used for RNA labelling, hybridization in the microarray and fluorescence signal scanning were performed as described before [24]. Briefly, 10 µg of total RNA collected from IST439 and IST4113 isolates was used for cDNA synthesis and subsequent labelling with Cy3 using the CyScribe Post-Labelling Kit (GE Healthcare) and following the manufacturer's recommendations. Genomic DNA of *B. cenocepacia* J2315 strain (1 µg) was labelled with Cy5 using the CyScribe Array CGH Genomic DNA Labeling System (GE Healthcare) and used as an internal control for the microarray hybridization. After hybridization and scanning, the microarray image was analysed using Agilent Feature Extraction software and only those probes considered “Present” by the software were selected for further analysis. Gene expression analysis was performed using GeneSpring GX 7.3 (Agilent) and data were normalized using the Affymetrix FE data normalization procedure recommended for two-colour Agilent microarrays. For each sample, the intensity measured for a given gene was divided by the value obtained in the control channel value. Only those genes whose transcript levels difference in IST439 and in IST4113 were equal or above 1.5-fold were considered to be differentially transcribed in the two variants. A one-way ANOVA analysis was performed over the gathered data and only the genes having an associated *p*-value below 0.05 were considered for further analysis. The raw data of the microarray analysis is MIAME compliant and was deposited in the Array Express database (E-MTAB-809).

### Quantitative RT-PCR

The transcript levels from a selected set of genes were compared in IST439 and IST4113 using quantitative RT-real time PCR (qRT-PCR). Bacterial growth and RNA extraction were performed as described above for the microarray analysis. Synthesis of cDNA was performed from 200 ng of total RNA using the Reverse Transcription kit from Applied Biosystems. The cDNA obtained was diluted (1∶4) in RNase-free water and used as template for the subsequent PCR step. This second step was carried out in a Thermal Cycler Block (Applied Biosystems) and SYBR® Green 7500 RT-PCR (Applied Biosystems) was used to accompany the increase in fluorescence during DNA synthesis. The primers used in gene amplifications were designed to exclude cross-hybridizations and their sequence is available upon request.

### Comparison of the intracellular accumulation of ethidium bromide in IST439 and IST4113 cells

The quantitative comparison of the intracellular accumulation of ethidium bromide inside IST439 and IST4113 cells was estimated using the ethidium bromide agar screening method [77]. Isolated colonies of IST439 and IST4113 were cultivated in LB liquid medium until mid-exponential phase (OD_640 nm_ of 0.4±0.05), diluted in NaCl 0.9% to obtain a cell suspension with an OD_640 nm_ of 0.2±0.05. These cell suspensions were spotted, in duplicate, onto the surface of LB plates supplemented with ethidium bromide (concentrations ranging from 0 to 1.0 mg/L) and the plates were incubated for 24 h at 37°C. One set of plates was incubated for an additional period of 4 h at 37°C while the other set was incubated for the same time at 4°C. The fluorescence emitted by the bacterial mass in the plate was assessed in a UV transilluminator (Bio-Rad Gel Doc XR System) and quantified using the Quantity One software V 4.5.2 (Bio-Rad Laboratories).

## Supporting Information

Table S1 **Sub-set of genes found to be differently transcribed (above or below 1.5-fold) in IST439 and IST4113.** The genes whose transcript levels in the IST4113 variant were above, or below, 1.5-fold the values attained in IST439 were considered to be differently expressed in the two variants and were selected for further analysis. The genes found to be down-regulated in IST4113 are shaded in grey. The full list of genes that have altered transcript levels in the two variants is provided in Supplementary Tables S1 and S2. These genes were grouped according with their biological function using the functional clustering indicated in Figure 1.
(PDF)

Table S2 **Full list of genes up-regulated (above 1.5-fold) in** ***B. cenocepacia*** **IST4113, compared to IST439.** The genes whose transcript level in the *B. cenocepacia* IST4113 variant were at least 1.5-fold higher than the levels attained in the IST439 were selected. The description of gene function is based on the information available in the “*Burkholderia* Genome Database” and in “KEGG Pathways” database and the functional clustering was performed using the functional classes shown in Figure 1. Genes specific of the AU1054 strain are shaded in grey.
(PDF)

Table S3 **Full list of genes down-regulated (above 1.5-fold) in** ***B. cenocepacia*** **IST4113, compared to IST439.** The genes whose transcript level in the *B. cenocepacia* IST4113 variant were at least 1.5-fold lower than the levels attained in IST439 were selected. The description of gene function is based on the information available in the “*Burkholderia* Genome Database” and in “KEGG Pathways” database and the functional clustering was performed using the functional classes shown in Figure 1. Genes specific of the AU1054 strain are shaded in grey.
(PDF)

Table S4 **List of genes having an altered expression in** ***B. cenocepacia*** **variants IST439 and IST4113 that are documented to be regulated by CciR.** The microarray dataset was searched for genes documented to be activated or repressed by the CciR transcription factor using the results of O'Grady *et al*., (2010).
(PDF)

Table S5 **Proteins found in the membrane-enriched fraction and in the cytosolic fraction whose content was increased (above 1.5-fold) or decreased (below 0.7-fold) in the proteome of** ***B. cenocepacia*** **IST4113 cells, compared to the values registered in** ***B. cenocepacia*** **IST439 (Madeira** ***et al*** **., 2011).** The genes predicted to encode these proteins are also indicated and those found to be differently expressed in the two variants, based on the results of the microarray analysis carried out in this study, are highlighted in bold.
(PDF)
