# Supplementary material for: Genomic Expression Analysis Reveals Strategies of Burkholderia cenocepacia to Adapt to Cystic Fibrosis Patients' Airways and Antimicrobial Therapy
Source: PLoS One. 2011 Dec 21;6(12):e28831. doi: 10.1371/journal.pone.0028831 (PMC3244429; doi:10.1371/journal.pone.0028831)
Supplement: Table S1 — Sub-set of genes found to be differently transcribed (above or below 1.5-fold) in IST439 and IST4113. The genes whose transcript levels in the IST4113 variant were above, or below, 1.5-fold the values attained in IST439 were considered to be differently expressed in the two variants and were selected for further analysis. The genes found to be down-regulated in IST4113 are shaded in grey. The full list of genes that have altered transcript levels in the two variants is provided in Supplementary Tables S1 and S2. These genes were grouped according with their biological function using the functional clustering indicated in Figure 1. (PDF) [file pone.0028831.s001.pdf]

Supplementary Table S1. Sub-set of genes found to be differently transcribed (above or below 1.5-fold) in IST439 and IST4113.

CDS/Gene	Function	mRNA IST4113 / mRNA IST439
Carbohydrate metabolism		
Electron transport and oxidative phosphorylation		
BCAL0030/*atpB*	F_0F_1 ATP synthase subunit A	1.7
BCAL0037/*atpC*	F_0F_1 ATP synthase subunit epsilon	1.7
BCAM2674	Putative cytochrome oxidase subunit I	2.7
BCAM2675	Putative cytochrome oxidase subunit II	1.5
BCAL0328/*petA*	Ubiquinol-cytochrome c reductase iron-sulfur subunit; involved in aerobic respiration	-1.9
BCAL0329/*petB*	Cytochrome *b* of the cytochrome *bc* complex; involved in aerobic respiration	-2.4
BCAL0330/*petC*	Cytochrome *c1* precursor	-1.6
BCAL0388	Ubiquinone biosynthesis hydroxylase family protein	-2.4
BCAL0784/*cydA*	Cytochrome *d* ubiquinol oxidase subunit II (cytochrome *bd*)	-1.6
BCAL0785/*cydB*	Cytochrome *d* ubiquinol oxidase subunit II (cytochrome *bd*)	-2.3
BCAL2141/*cyoD*	Cytochrome *o* ubiquinol oxidase	-2.2
BCAL2142/*cyoC*	Cytochrome *o* ubiquinol oxidase subunit III	-2.1
BCAL2143/*cyoB*	Ubiquinol oxidase polypeptide I	-2.6
BCAL2144/*cyoA*	Ubiquinol oxidase polypeptide II precursor	-2.3
Glyoxylate cycle		
BCAL2122/*aceB*	Malate synthase	1.6
BCAM1588	Isocitrate lyase	2.3
Acetoin catabolism		
BCAL1909/*acoC*	Branched-chain alpha-keto acid dehydrogenase subunit E2	4.8
BCAL1910/*acoB*	Acetoin:2,6-dichlorophenolindophenol oxidoreductase beta subunit	1.8
BCAL1911/*acoA*	Acetoin:2,6-dichlorophenolindophenol oxidoreductase alpha subunit	2.5
BCAL1913/*acoX*	Acetoin catabolism protein	3.6
Amino acid and nitrogen metabolism		
Ammonium metabolism		
BCAL2224/*glnA*	Glutamine synthetase; involved in ammonium reduction	1.6
BCAM2301	Putative methylamine dehydrogenase;	2.3
BCAM2302A	Putative methylamine utilization protein	2.2
BCAS0114	Putative histidine-ammonia lyase	1.5
BCAM2304	Putative methylamine dehydrogenase	1.9

CDS/Gene	Function	mRNA IST4113 / mRNA IST439
Glycine catabolism		
BCAL0075/*gcvT*	Glycine cleavage system aminomethyltransferase T; involved in ammonium reduction	2.4
BCAL0073/*gcvP*	Glycine dehydrogenase	2.0
BCAL0074/*gcvH*	Glycine cleavage system protein H	2.7
Histidine catabolism		
BCAL2240/*hutG*	N-formylglutamate deformylase	1.9
BCAL2241	N-formimino-L-glutamate deiminase	1.5
BCAL2244/*hutU*	Urocanate hydratase	1.5
Aspartate, glutamate and glutamine catabolism		
BCAL2146/*argT*	Argininosuccinate synthase involved in arginine biosynthesis	1.5
BCAM0746/*ask*	Aspartate kinase	2.9
BCAL0600	Putative glutamine synthetase	2.4
Phenylalanine catabolism		
BCAM0810	Putative aromatic oxygenase	1.5
BCAM0811	Putative aromatic oxygenase	1.7
BCAM1315/*amiE*	Acylamide amidohydrolase; involved in putrescine biosynthesis	2.7
BCA1979/*feaB*	Phenylacetaldehyde dehydrogenase	1.5
Sulphur metabolism		
BCAM1121	Putative taurine dioxygenase	3.7
BCAM1122	Putative taurine dioxygenase	4.1
BCAM1676	Putative nitrite/sulfite reductase that reduces sulfite into H_2S; involved in cysteine biosynthesis	1.6
BCAM1391	Putative alkanesulfonate monooxygenase which convert alkanesulfonates into sulfite	1.6
BCAM1565	Putative alkanesulfonate monooxygenases	1.9
BCAL1619/*ssuD*	Putative alkanesulfonate monooxygenases	1.7
BCAM0557	Putative alkanesulfonate monooxygenases	3.4
BCAS0054	Putative alkanesulfonate monooxygenases	4.5
BCAL2049	Putative oxidoreductase presumed to be involved in conversion of adenylnylsulfate into sulfite	1.8
BCAL2681/*cysN*	Putative sulfate adenylyltransferase subunit 1	-1.6
BCAL2682/*cysD1*	Sulfate adenylyltransferase subunit 2	-1.8
BCAL2683/*cysH*	Phosphoadenosine phosphosulfate reductase	-1.5

CDS/Gene	Function	mRNA IST4113 / mRNA IST439
Transport		
Sulphur-based compounds		
BCAL0711/*tauA*	Periplasmic component of the ABC taurine transporter TauABC	3.2
BCAL0712/*tauB*	ATPase componenent of the ABC taurine transporter TauABC	2.2
BCAL0713/*tauC*	Permease component of taurine ABC transporter TauABC	2.5
BCAL1620/*ssuC*	Permease component of the ABC transporter SsuABC required for uptake of aliphatic sulfonates	1.8
BCAL1621/*ssuB*	ATPase component of of the ABC transporter SsuABC required for uptake of aliphatic sulfonates	1.7
Sugars		
BCAL1377	H^+/citrate symporter	1.8
BCAL1431	Putative outer membrane component of an ABC transport system required for ribose uptake	1.8
BCAL0781	Phosphotransferase system IIC components; specific for glucose/maltose/N-acetylglucosamine	3.2
BCAL1092	Periplasmic component of a ABC transporter similar to a 2-aminoethylphosphonate transport system	1.7
BCAM0153/*kgdT*	2-keto-3-deoxygluconate permease	1.8
BCAM2509	Putative fucose transporter	1.6
BCAM1986	Putative sugar transporter of the Major Facilitator Superfamily	1.9
BCAM2545	Putative sugar transporter of the Major Facilitator Superfamily	2.5
BCAM2697	Putative sugar transporter of the Major Facilitator Superfamily	2.1
BCAS0122	Putative sugar transporter of the Major Facilitator Superfamily	1.6
BCAS0343	Putative sugar transporter of the Major Facilitator Superfamily	1.8
BCAS0438	Predicted periplamic component of an ABC sugar transporter	1.9
Amino acids and peptides		
BCAM0978/*lysP*	Lysine-specific permease	1.7
BCAL1668	Predicted periplasmic component of a putative arginine/ornithine/lysine ABC transporter	1.9
BCAL0766	Putative branched-chain amino acid transport system permease protein	2.0
BCAL1252	Putative proline/betaine transporter	2.2
BCAL2729	Putative amino acid permease predicted to be involved in uptake of GABA	1.9
BCAM2248/*livG*	ATPase component of a leucine/isoleucine/valine amino acid ABC transporter	2.0

CDS/Gene	Function	mRNA IST4113 / mRNA IST439
BCAM2249/*livM*	Permease component of a leucine/isoleucine/valine amino acid ABC transporter	1.9
BCAM2618/*argT*	Periplasmic component of a putative arginine/ornithine/lysine ABC transporter	1.9
BCAM2724/*oppF*	ATPase component of Putative oligopeptide ABC transporter	1.8
BCAM2725/*oppD*	ATPase component of Putative oligopeptide ABC transporter	2.0
BCAM2726/*oppC*	Permease component of Putative oligopeptide ABC transporter	1.7
BCAS0110	Predicted periplasmic component of a putative arginine/ornithine/lysine ABC transporter	1.6
Cell envelope and outer membrane		
LPS and peptidoglycan biosynthesis		
BCAL2180	3-deoxy-8-phosphooctulonate synthase presumed to be involved in synthesis of 3-deoxy-D-manno-octulosonic acid; required for LPS biosynthesis	1.5
BCAL0779	Putative phosphosugar-binding protein presumed to be involved in peptidoglycan synthesis	3.4
BCAL2759	Tetraacyldisaccharide-1-P-kinase involved in lipid A biosynthesis	2.3
BCAM0214	Putative glycosyltransferase presumed to be involved in LPS biosynthesis	2.9
BCAM0220	Putative outer membrane protein presumed to be involved in the secretion of peptidoglycan-associated (lipo)proteins	2.3
BCAL0067	ADP-heptose:LPS heptosyltransferase	-1.6
BCAL2205	D-alanyl-D-alanine endopeptidase (penicillin-binding protein precursor)	-1.6
BCAL3110/*waaA*	3-deoxy-D-manno-octulosonic-acid transferase involved in biosynthesis of O-antigen	-1.6
BCAL3111/*wbxY*	Protein of unknown function that is involved in biosynthesis of O-antigen	-1.6
BCAM2067/*uppS*	Putative undecaprenyl pyrophosphate synthetase involved in peptidoglycan biosynthesis	-3.7
Protein secretion		
BCAL1671	Metallo peptidase	2.5
BCAM0180	Metallo peptidase	3.3
BCAL2466/*eco*	Serine-protease inhibitor ecotin	1.8
BCAL2867/*lepA*	GTP-binding protein that functions as an exoprotease	1.5
BCAM0550	Putative exported lipase	1.9
Adhesion		
BCAM0184/*bclB*	Lectin; required for attachment to mucin	1.7

CDS/Gene	Function	mRNA IST4113 / mRNA IST439
BCAM0185/*bclC*	Lectin; required for attachment to mucin	1.5
BCAM0219	Putative haemagglutinin-related autotransporter protein	1.5
BCAM0223	Putative haemagglutinin-related autotransporter protein	3.8
BCAL1086/*bclA*	Lectin; required for attachment to mucin	-2.1
BCAM0224	Putative haemagglutinin autotransporter protein	-1.6
Protein synthesis		
BCAL0115/*rpsU1*	Structural component of the ribosome	2.0
BCAL0222/*rplK*	50S ribosomal protein L11	1.7
BCAL0224/*rplJ*	50S ribosomal protein L10	2.2
BCAL0225/*rplL*	50S ribosomal protein L7/L12	2.4
BCAL0229/*rpsL*	30S ribosomal protein S12	1.9
BCAL230/*rpsG*	30S ribosomal protein S12	1.8
BCAL0233/*rpsJ*	30s ribosomal protein S10	1.6
BCAL0234/*rplC*	50S ribosomal protein L3	2.3
BCAL0235/*rplD*	50S ribosomal protein L4	2.8
BCAL0236/*rplW*	50S ribosomal protein L23	2.5
BCAL0237/*rplB*	50S ribosomal protein L2	2.9
BCAL0238/*rpsS*	30S ribosomal protein S19	2.7
BCAL0239/*rplV*	50S ribosomal protein L22	2.8
BCAL0240/*rpsC*	30S ribosomal protein S3	2.9
BCAL0241/*rplP*	50S ribosomal protein L16	2.0
BCAL0242/*rpmC*	50S ribosomal protein L29	2.3
BCAL0243/*rpsQ*	30S ribosomal protein S17	2.6
BCAL0244/*rplN*	50S ribosomal protein L14	1.8
BCAL0245/*rplX*	50S ribosomal protein L24	1.5
BCAL0246/*rplE*	50S ribosomal protein L5	1.8
BCAL0247/*rpsN*	30S ribosomal protein S14	2.2
BCAL0248/*rpsH*	30S ribosomal protein S8	2.1
BCAL0249/*rplF*	50S ribosomal protein L6	1.9
BCAL0250/*rplR*	50S ribosomal protein L18	2.1
BCAL0251/*rpsE*	30S ribosomal protein S5	1.9
BCAL0252/*rpmD*	50S ribosomal protein L30	1.7
BCAL0253/*rplO*	50S ribosomal protein L15	1.9
BCAL0256/*rpmJ*	50S ribosomal protein L36	1.8
BCAL0257/*rpsM*	30S ribosomal protein S13	1.9
BCAL0258/*rpsK*	30S ribosomal protein S11	2.2
BCAL0259/*rpsD*	30S ribosomal protein S4	1.7
BCAL0261/*rplQ*	50S ribosomal protein L17	1.5
BCAL0799	50S ribosomal protein L25/general stress protein Ctc	1.9
BCAL0990/*rpmF*	50S ribosomal protein L32	1.9
BCAL1483/*rpmI*	50S ribosomal protein L35	2.3
BCAL1484/*rpiT*	50S ribosomal protein L20	2.2
BCAL1905/*rpmE2*	50S ribosomal protein L31 type B	2.7
BCAL1942/*rplI*	50S ribosomal protein L9	2.0

BCAL1943/*rpsR*	30S ribosomal protein S18	1.8
BCAL1945/*rpsF*	30S ribosomal protein S6	1.8
BCAL2715/*rpmG*	50S ribosomal protein L33	1.5
BCAL2765/*rpsT*	30S ribosomal protein S20	1.6
BCAL2928/*rpsP*	30S ribosomal protein S16	1.6
BCAL2950/*rpsA*	30S ribosomal protein S1	1.7
BCAL3347/*rpsI*	30S ribosomal protein S9	1.6
BCAL3348/*rplM*	50S ribosomal protein L13	1.7
BCAL3442/*rplU*	50S ribosomal protein L21	1.6
BCAL0219/*tufA1*	Elongation factor Tu	1.8
BCAL0231/*fusA*	Elongation factor G	2.5
BCAL0232/*tuf*	Elongation factor Tu	1.8
BCAl1012/*efp2*	Elongation factor P	1.9
BCAL0255/*infA*	Translation initiation factor IF-1	2.1
BCAL1507/*infB*	Translation initiation factor IF-2	1.8
<u>Ion homeostasis</u>		
Iron assimilation		
BCAL1697/*orbJ*	Non-ribosomal peptide synthase required for ornibactin biosynthesis	1.9
BCAL1698/*orbK*	Protein of unknown function involved in ornibactin biosynthesis	3.0
BCAL1699/*pvdA*	L-ornithine-oxygenase involved in ornibactin biosynthesis	4.9
BCAL1700/*orbA*	Ornibactin receptor; presumably involved in the transport of iron-ornibactin complexes	5.9
BCAL1701/*orbF*	Ornibactin synthetase	4.1
BCAL1702/*orbL*	Protein of unknown function involved in ornibactin biosynthesis	3.1
BCAM2626/*huvA*	Putative heme receptor protein	2.0
BCAM2627/*hmuS*	Putative hemin ABC transport system protein	1.7
BCAM0564	TonB-dependent receptor	1.5
BCAL1371	TonB-dependent receptor	1.6
BCAM1571	TonB-dependent receptor	1.9
BCAM2227/*phcG*	Putative pyochelin biosynthetic protein	-1.6
BCAM2230/*phcE*	Dihydroaeruginoic acid synthetase PchE involved in pyochelin biosynthesis	-1.5
