# Supplementary material for: Genomic Expression Analysis Reveals Strategies of Burkholderia cenocepacia to Adapt to Cystic Fibrosis Patients' Airways and Antimicrobial Therapy
Source: PLoS One. 2011 Dec 21;6(12):e28831. doi: 10.1371/journal.pone.0028831 (PMC3244429; doi:10.1371/journal.pone.0028831)
Supplement: Table S2 — Full list of genes up-regulated (above 1.5-fold) in B. cenocepacia IST4113, compared to IST439. The genes whose transcript level in the B. cenocepacia IST4113 variant were at least 1.5-fold higher than the levels attained in the IST439 were selected. The description of gene function is based on the information available in the “Burkholderia Genome Database” and in “KEGG Pathways” database and the functional clustering was performed using the functional classes shown in Figure 1. Genes specific of the AU1054 strain are shaded in grey. (PDF) [file pone.0028831.s002.pdf]

Table S1 - List of genes up-regulated (above 1.5-fold) in *B. cenocepacia* IST4113, compared to IST439. The genes whose transcript level in the *B. cenocepacia* IST4113 variant were at least 1.5-fold higher than the levels attained in the IST439 were selected. The description of gene function is based on the information available in the "*Burkholderia* Genome Database" and in "KEEG pathways" database and the functional clustering was performed using the functional classes shown in Figure 1. Genes specific of the AU1054 strain are shaded in grey

ORF/Gene	Function	mRNA IST4113 / mRNA IST439
Carbohydrate metabolism		
Electron transport and oxidative phosphorylation		
BCAL0030/atpB	F0F1 ATP synthase subunit A	1.6
BCAL0037/atpC	F0F1 ATP synthase subunit epsilon	1.7
BCAL2050	4Fe-4S ferredoxin	1.6
BCAM0166/ndh	NADH dehydrogenase	3.0
BCAM0182	Putative cytochrome b561	2.4
BCAM0692	Putative NADPH-dependent FMN reductase	1.5
BCAM1386	Putative cytochrome c	2.0
BCAM2299	Putative cytochrome c	2.5
BCAM2300	Putative cytochrome c	2.3
BCAS0417	Putative cytochrome c	1.9
BCAM2674	Putative cytochrome oxidase subunit I	2.7
BCAM2675	Putative cytochrome oxidase subunit II	1.5
BCAM1961	Copper binding protein; similar to plastocyanine	1.9

Biosynthesis of vitamines and coenzymes		
BCAL0599	Putative aminotransferase presumed to be involved in biotin synthesis	3.6
BCAS0383	Putative adenosylmethionine-8-amino-7-oxononanoate transaminase involved in biothin biosynthesis	2.6
BCAM1978	Adenosylmethionine-8-amino-7-oxononanoate aminotransferase presumed to be involved in biotin biosynthesis	2.1
BCAL0915	Putative 3,4-dihydroxy-2-butanone 4-phosphate synthase involved in riboflavin biosynthesis from ribulose-1,5-biphosphate	1.8
BCAL0206	Indolepyruvate ferredoxin oxidoreductase that catalyses the oxidative decarboxylation of arylpyruvates	2.6
BCAL2717/nadC	Nicotinate-nucleotide pyrophosphorylase, required for NAD biosynthesis	1.6
BCAL2718/nadA	Quinolinate synthetase, required for NAD biosynthesis	1.7
BCAL3050/ribD	Multifunctional riboflavin biosynthetic protein (includes: diaminohydroxyphosphoribosylaminopyrimidine deaminase; 5-amino-6-(5-phosphoribosylamino)uracil reductase)	1.6
BCAL3051/ribE	Riboflavin synthase subunit alpha	1.5
BCAL3049/hemL	Glutamate-1-semialdehyde 2,1-aminomutase involved in porphyrin biosynthesis	1.5
Glyoxylate cycle		
BCAL2122/aceB	Malate synthase; required for the biosynthesis of malate from glyoxylate and acetyl-CoA	1.6
BCAM1588	Isocitrate lyase	2.3
Acetoin catabolism		
BCAL1908/lipA	Putative lipoate synthase involved in lipoic acid biosynthesis	3.5
BCAL1909/acoC	Branched-chain alpha-keto acid dehydrogenase subunit E2	4.8
BCAL1910/acoB	Acetoin:2,6-dichlorophenolindophenol oxidoreductase beta subunit	1.8
BCAL1911/acoA	Acetoin:2,6-dichlorophenolindophenol oxidoreductase alpha subunit	2.5
BCAL1913/acoX	Acetoin catabolism protein	3.6
BCAL1225	Putative aminohydrolase similar to hydrolases involved in acetoin catabolism	1.7

Benzoate catabolism		
BCAS0494/benC	Benzoate 1,2-dioxygenase electron transfer component	2.2
BCAS0495/benB	Benzoate 1,2-dioxygenase beta subunit	2.0
BCAS0496/benA	Benzoate 1,2-dioxygenase alpha subunit	1.6
BCAS0497/catA	Catechol 1,2-dioxygenase 2	2.8
BCAM0606	Related to carboxymuconolactone decarboxylases; predicted to function in benzoate degradation	2.4
BCAL1775	Reductase component of dioxygenase system predicted to be involved in degradation of aminobenzoates	2.0
Others		
BCAL3285/hmpA	Flavohemoprotein presumed to be involved in phenylacetate catabolism	1.5
BCAS0053/msuE	FMN reductase	1.6
BCAM2122	Acetaldehyde dehydrogenase that converts acetyl-CoA into acetaldehyde	3.1
BCAL0780	Putative multiphosphoryl transfer protein predicted to be involved in phosphorylation of fructose and phosphoenolpyruvate	1.8
BCAM0551	Similar to ribulose-5-phosphate 4-epimerase and related epimerases and aldolases	1.6
BCAL0778	Putative N-acetylglucosamine-6-phosphate deacetylase	3.2
**Aminoacid and nitrogen metabolism**		
Ammonium metabolism		
BCAL2224/glnA	Glutamine synthetase	1.6
BCAL0600	Putative glutamine synthetase	2.4
BCAM2301	Putative methylamine dehydrogenase	2.3
BCAM2302A	Putative methylamine utilization protein	2.2
BCAM2304	Putative methylamine dehydrogenase	1.9
Glycine catabolism		
BCAL0075/gcvT	Glycine cleavage system aminomethyltransferase T	2.4
BCAL0073/gcvP	Glycine dehydrogenase	2.0

BCAL0074/gcvH	Glycine cleavage system protein H	2.7
Histidine catabolism		
BCAL2240/hutG	N-formylglutamate deformylase	1.9
BCAL2241	N-formimino-L-glutamate deiminase	1.5
BCAL2244/hutU	Urocanate hydratase	1.5
BCAS0114	Putative histidine ammonia-lyase	1.5
Aspartate, glutamate and glutamine metabolism		
BCAM0746/argG	argininosuccinate synthase involved in arginine biosynthesis	1.5
BCAL2146/ask	aspartate kinase involved in aspartate catabolism	2.9
Phenylalanine metabolism		
BCAM1315/amiE	Acylamide amidohydrolase presumed to be involved in putrescine biosynthesis	2.7
BCAM0810	Putative aromatic oxygenase presumed to be involved in phenylalanine catabolism	1.5
BCAM0811	Putative aromatic oxygenase presumed to be involved in phenylalanine catabolism	1.7
BCAM1979/feaB	Phenylacetaldehyde dehydrogenase	1.5
Others		
BCAM1704/adh	2,3-butanediol dehydrogenase involved in threonine catabolism	5.3
BCAM1151	Methylmalonate-semialdehyde dehydrogenase; involved in valine catabolism	1.7
BCAL2354/leuA	2-isopropylmalate synthase involved in leucine biosynthesis	1.6
BCAM0540	Putative serine acetyltransferase that converts serine in acetyl-serine; involved in biosynthesis of cysteine	1.6
BCAM0976	Putative amidinotransferase predicted to be involved in citrullin biosynthesis	2.5
BCAL1796	Predicted saccharopine dehydrogenase involved in lysine biosynthesis	1.5
BCAS0211	Putative pyridoxal-dependent decarboxylase presumed to be involved in lysine biosynthesis	1.5
BCAL1926/hom	Homoserine dehydrogenase; involved in formation of homoserine	1.6
BCAL0603/puuC	γ-glutamyl-gamma-aminobutyraldehydedehydrogenase	1.7
BCAM0543	Putative aminotransferase presumed to be involved in the interconversion of alanine and selenocistheyne	1.5

BCAS0381/gabD	succinate-semialdehyde dehydrogenase involved in formation of succinate from glutamate	1.5
BCAL3282/aroG	phospho-2-dehydro-3-deoxyheptonate aldolase involved in biosynthesis of chorismate (a precursor of aromatic amino acids) from phosphoenolpyruvate	1.6
**Purine and pyrimidine metabolism**		
BCAL1872	Putative nucleotide phosphoribosyltransferase	1.5
BCAL1887	Nucleoside diphosphate kinase involved in conversion of Inosinate triphosphate to inosinate biphosphate	1.7
BCAL2061/guaA	GMP synthase	1.7
BCAL2063/guaB	Inosine 5'-monophosphate dehydrogenase that converts inosine monophosphate into xanthosine monophosphate	1.5
BCAL3138/pyrX	Dihydroorotase that converts carbamoyl aspartic acid into 4,5-dihydroorotic acid in pyrimidine biosynthesis	1.6
BCAL0204/pyrE	Orotate phosphoribosyltransferase that converts orotate into orotidine-5-phosphate in pyrimidine biosynthesis	1.5
Lipid metabolism		
BCAL0391/acpD	Acyl carrier protein phosphodiesterase	2.4
BCAL1435/idh	Inositol 2-dehydrogenase	2.1
BCAL1436	Putative dehydratase presumed to be involved in inositol phosphate metabolism	1.7
BCAL1862/phbA	Acetyl-CoA acetyltransferase	1.5
BCAL2725/lspA	Lipoprotein signal peptidase	1.5
BCAM0171	Putative fatty acid desaturase	2.0
BCAM1582	3-alpha-(or 20-beta)-hydroxysteroid dehydrogenase involved in fatty acid biosynthesis	1.6
BCAM2526	Putative monooxygenase similar to Acyl-CoA dehydrogenases	1.8
BCAS0346	Putative dehydrogenase similar to acyl-CoA dehydrogenases	2.0
BCAS0419	Putative acyl-CoA dehydrogenase protein	1.6
Sulphur metabolism		
BCAM1121	Putative taurine dioxygenase (similar to TauD) that converts taurine into sulfite	3.7
BCAM1122	Putative taurine dioxygenase (similar to TauD) that converts taurine into sulfite	4.1
BCAS0426	Putative taurine dioxygenase (similar to TauD) that converts taurine into sulfite	1.6

BCAM1676	Putative nitrite/sulfite reductase that reduces sulfite into H2S; involved in biosynthesis of L-cysteine	1.6
BCAM1391	Putative alkanesulfonate monooxygenase which convert alkanesulfonates into sulfite	1.6
BCAM1565	Putative alkanesulfonate monooxygenase which reduce alkanesulfonates into sulfite	1.9
BCAL1619/ssuD	Putative alkanesulfonate monooxygenase which reduce alkanesulfonates into sulfite	1.7
BCAM0557	Putative alkanesulfonate monooxygenase which reduce alkanesulfonates into sulfite	3.4
BCAS0054	Putative alkanesulfonate monooxygenase which reduce alkanesulfonates into sulfite	4.5
BCAL2049	Putative oxidoreductase presumed to be involved in conversion of adenylnylsulfate into sulfite	1.7

Transport

Sulphur-based compounds

BCAL0711/tauA	Periplasmic component of the ABC taurine transporter TauABC	3.2
BCAL0712/tauB	ATPase componenent of the ABC taurine transporter TauABC	2.2
BCAL0713/tauC	Permease component of taurine ABC transporter TauABC	2.5
BCAL1620/ssuC	Permease component of the ABC transporter of aliphatic sulfonates SsuABC	1.8
BCAL1621/ssuB	ATPase component of the ABC transporter of aliphatic sulfonates SsuABC	1.7
BCAL2051	Periplasmic component of the Putative alkanesulfonates ABC transporter SsuABC	1.6
BCAL2052	Permease component of a Putative alkanesulfonates ABC transporter	1.9
BCAL2053	ATPase component of a Putative alkanesulfonates ABC transporter	1.6
BCAM0555	Periplasmic component of a Putative alkanesulfonates ABC transporter	2.1
BCAM0556	Periplasmic component of a Putative alkanesulfonates ABC transporter	2.8
BCAM0558	Periplasmic component of a Putative alkanesulfonates ABC transporter	3.4
BCAM1123	ATPase component of a Putative alkanesulfonates ABC transporter	3.0
BCAM1124	Permease component of a Putative alkanesulfonates ABC transporter	2.4
BCAM1125	Permease component of a Putative alkanesulfonates ABC transporter	2.0
BCAM1126	Periplasmic component of a Putative alkanesulfonates ABC transporter	2.2
BCAM1377	Predicted ATPase component of a sulfate ABC transporter; similar to PotA which is involved in the uptake of spermidine and putrescine	2.1
BCAM1378	Predicted substrate-binding component of a sulfate ABC transporter	2.1
BCAM1379	Predicted periplasmic component component of a sulfate ABC transporter	1.5

BCAM1387	ATPase component of a Putative sulfonate ABC transporter	1.5
BCAM1389	Periplasmic component of a Putative sulfonate ABC transporter	1.9
BCAM1566	Periplasmic component of a Putative sulfonate ABC transporter	2.0
BCAS0340	ATPase component of a Putative sulfonate ABC transporter	1.9
BCAS0341	Permease component of a Putative alkanesulfonates ABC transporter	1.5
BCAS0342	Periplasmic component of a Putative sulfonate ABC transporter	1.8
BCAS0425	Predicted periplasmic component of an alkanesulfonate transporter	2.2
Polyamines		
BCAL0595/potI	Permease component II of the PotABCD ABC transporter involved in the uptake of spermidine and putrescine	2.7
BCAL0596/potH	Permease component I of the PotABCD ABC transporter involved in the uptake of spermidine and putrescine	1.7
BCAL0598/potF	Spermidine/putrescine-binding periplasmic protein; subunit of the PotABCD transporter involved in the uptake of spermidine and putrescine	2.7
Drugs		
BCAL1079/mdtC	Subunit of the multidrug resistance transporter MdtABC of the RND superfamily	1.6
BCAL1080/mdtB	Subunit of the multidrug resistance transporter MdtABC of the RND superfamily	1.6
BCAL1081/mdtA	Subunit of the multidrug resistance transporter MdtABC of the RND superfamily	1.7
BCAL2822/bpeA	Multidrug resistance transporter of the RND family	1.6
BCAM0201	Similar to the multidrug resistance transporter EmrB from E. coli	1.6
BCAM2188	Outer membrane protein; component of a predicted macrolide-specific efflux system	2.2
Aminoacids and peptides		
BCAM0978/lysP	Lysine-specific permease	1.7
BCAL1668	Predicted periplasmic component of a Putative arginine/ornithine/lysine ABC transporter	1.9
BCAL0019	Predicted ATPase component of a Putative aminoacid ABC transporter	1.6
BCAL0151	Predicted periplamic component of an ABC aminoacid transporter	1.5
BCAL0766	Putative branched-chain amino acid transport system permease protein	2.0
BCAL1252	Putative proline/betaine transporter	2.2
BCAL1423	Predicted ATPase component of a Putative aminoacid ABC transporter	2.0
BCAL2729	Putative amino acid permease predicted to be involved in uptake of GABA	1.9

BCAM0610	Predicted periplamic component of an ABC aminoacid transporter	1.6
BCAM0611	Predicted permease component of a Putative aminoacid ABC transporter	1.7
BCAM0612	Predicted ATPase component of a Putative aminoacid ABC transporter	1.5
BCAM1219	Predicted periplamic component of an ABC aminoacid transporter	1.5
BCAM1427	Putative Homoserine/Threonine efflux protein	5.3
BCAM1449A	Putative amino acid permease	1.7
BCAM1560	Predicted periplasmic component of a Putative arginine/ornithine/lysine ABC transporter	1.5
BCAM1563	Predicted ATPase component of a Putative aminoacid ABC transporter	2.2
BCAM1564	Predicted periplasmic component of a Putative aminoacid ABC transporter	1.9
BCAM2247	ATPase component of a Putative branched aminoacid ABC transporter	2.1
BCAM2248/livG	ATPase component of a leucine/isoleucine/valine aminoacid ABC transporter	2.0
BCAM2249/livM	Permease component of a leucine/isoleucine/valine aminoacid ABC transporter	1.9
BCAM2618/argT	Periplasmic component of a Putative arginine/ornithine/lysine ABC transporter	1.9
BCAM2250	Predicted permease component of a Putative aminoacid ABC transporter	1.6
BCAM2251	Predicted periplasmic component of a Putative aminoacid ABC transporter	1.8
BCAM2316	Putative amino acid permease	1.5
BCAM2529	ATPase component of a Putative methionine transporter	1.7
BCAM2724/oppF	ATPase component of Putative oligopeptide ABC transporter	1.8
BCAM2725/oppD	ATPase component of Putative oligopeptide ABC transporter	2.0
BCAM2726/oppC	Permease component of Putative oligopeptide ABC transporter	1.7
BCAS0110	Predicted periplasmic component of a Putative arginine/ornithine/lysine ABC transporter	1.6
BCAS0136	Putative amino acid permease	4.6
BCAS0574	Predicted ATPase component of a Putative aminoacid ABC transporter	1.5
BCAS0575	Predicted permease component of a Putative aminoacid ABC transporter	1.6
Bcen_1448	lysine exporter protein; similar to LysE and YggA	2.9
Ions		
BCAL0055	Putative heavy metal resistance membrane ATPase	1.5
BCAM0238	Putative ion transporter	2.4
BCAM0439	Putative cation efflux system; similar to CzcD which mediates efflux of Co/Zn/Cd	1.6
BCAM1770	Predicted periplasmic component of a Putative nickel ABC transporter	1.8
BCAM1771	Predicted ATPase component of a Putative nickel ABC transporter	1.7
BCAM1772	Predicted permease component of a Putative nickel ABC transporter	2.1
BCAM1773	Predicted ATPase component of a Putative nickel ABC transporter	2.1

BCAM1774	Predicted ATPase component of a Putative nickel ABC transporter	1.6
BCAS0388	Predicted periplasmic component of a dipeptide/oligopeptide/nickel transporter	2.7
BCAS0389	Predicted permease component of a dipeptide/oligopeptide/nickel transporter	1.9
BCAS0390	Predicted permease component of a dipeptide/oligopeptide/nickel transporter	2.4
BCAS0391	Predicted ATPase component of a dipeptide/oligopeptide/nickel transporter	2.8
BCAM0712/czcB	Putative cobalt-zinc-cadmium transporter	1.5
BCAM0560	Similar to E. coli TolQ which is a subunit of the TolQRA complex that are involved in the maintenaince of outer membrane permeability and are required for the uptake of iron siderophores	2.7
BCAM0561	Similar to E. coli TolR which is a subunit of the TolQRA complex that are involved in the maintenaince of outer membrane permeability and are required for the uptake of iron siderophores	3.4
Sugars		
BCAL1377	H+/citrate symporter	1.8
BCAL1431	Putative ribose ABC transport system, substrate-binding exported protein	1.8
BCAL0781	Phosphotransferase system IIC components; specific for glucose/maltose/N-acetylglucosamine	3.2
BCAM0153/kgdT	2-keto-3-deoxygluconate permease	1.8
BCAL1092	Periplasmic component of a ABC transporter similar to a 2-aminoethylphosphonate transport system	1.7
BCAM2509	Putative fucose transporter	1.6
BCAL0640	Major Facilitator Superfamily protein; similar to sugar permeases	1.6
BCAL1223	Major Facilitator Superfamily protein; similar to arabinose efflux permeases	1.6
BCAM0036	Major Facilitator Superfamily protein; similar to sugar phosphate permeases	2.7
BCAM0441	Major Facilitator Superfamily protein; similar to arabinose efflux permeases	1.9
BCAM0563	Major Facilitator Superfamily protein similar to arabinose efflux permeases	2.2
BCAM0098	Permease component of a Putative ABC sugar transporter	1.9
BCAM2806	ATPase component of a Putative ABC sugar transporter	1.5
BCAM1986	Major Facilitator Superfamily protein; similar to sugar permeases	1.9
BCAM2545	Major Facilitator Superfamily protein; similar to d-galactonate transporters	2.5
BCAM2697	Major Facilitator Superfamily protein; similar to d-galactonate transporters	2.1
BCAS0122	Similar to sugar phosphate permeases	1.6
BCAS0343	Major Facilitator Superfamily protein; similar to sugar transporters	1.8

BCAS0438	Predicted periplamic component of an ABC sugar transporter	1.9
Ectoine		
BCAL1669	Predicted permease component of a Putative ectoine ABC transporter	1.8
BCAL1670	Predicted permease component of a Putative ectoine ABC transporter	1.8
BCAS0113	Predicted ATPase component of a Putative ectoine ABC transporter	1.5
Others		
BCAM1286	Putative betaine/carnitine/choline family transporter	1.6
BCAS0420	Predicted permease component of a Putative nitrate ABC transporter	1.5
Cell envelope and outer membrane		
LPS biosynthesis		
BCAL2759	Tetraacyldisaccharide-1-P 4'-kinase; predicted to be involved in LPS biosynthesis	2.2
BCAL2180	3-deoxy-8-phosphooctulonate synthase presumed to be involved in synthesis of 3-deoxy-D-manno-octulosonic acid	1.5
BCAM0214	Putative glycosyltransferase presumed to be involved in LPS biosynthesis	2.9
Peptidoglycan biosynthesis		
BCAL0110	Predicted pyridoxal phosphate-dependent enzyme apparently involved in regulation of cell wall biogenesis	1.6
BCAL0482/mreB	Rod shape-determining protein; presumed to be involved in the synthesis of the insertion of new peptidoglycan building units into the existing peptidoglycan layer to allow length growth	1.9
BCAL0779	Putative phosphosugar-binding protein presumed to be involved in peptidoglycan synthesis	3.4
BCAM2783/pehC	Endopygalactorunase	1.7
BCAM0220	Putative outer membrane protein presumed to be involved in the secretion of peptidoglycan-associated (lipo)proteins	2.3
Adhesion		
BCAM0184	Lectin	1.7
BCAM0185	Lectin	1.5

BCAM0219	Putative haemagglutinin-related autotransporter protein	1.5
BCAM0223	Putative haemagglutinin-related autotransporter protein	3.8
Others		
BCAL1671	Metallo peptidase, subfamily M23B	2.5
BCAM0180	metallo peptidase, subfamily M23B	3.3
BCAL2466/eco	ecotin	1.8
BCAL2867/lepA	GTP-binding protein proposed to function as an exoprotease	1.5
BCAM0550	Putative exported lipase	1.9
BCAL0906/Int	Apolipoprotein N-acyltransferase	2.3
BCAM0207	Putative tyrosine-protein kinase; predicted to be involved in exopolysaccharide biosynthesis	1.6
BCAM1376	Putative outer membrane porin; similar to OmpC	2.5
BCAM1455	Putative outer membrane porin; similar to OmpC	1.9
BCAS0066	Putative outer membrane porin; similar to OmpC; 35% similar to the probable TonB-dependent receptor PA3268	3.1
BCAM1974	Putative outer membrane porin; similar to OmpC	2.3
BCAM1761	Putative lipoprotein	1.5
BCAM0121	Putative lipoprotein	2.0
BCAL3438	Putative lipoprotein	1.7
BCAM1988	Putative lipoprotein	1.6
BCAS0305	Putative lipoprotein	2.2
BCAL0159	Putative lipoprotein	1.6
BCAS0625	Putative lipoprotein	1.6
BCAM0382	Putative lipoprotein	1.6
BCAL2166	Putative lipoprotein	1.9
BCAL1413A	Putative lipoprotein	1.7
BCAM1678	Putative lipoprotein; similar to bacterial virulence protein VirJ	1.8
BCAM2620	Putative lipoprotein	2.0
BCAS0627	TPR repeat, SEL1 subfamily	1.6

Protein synthesis		
BCAL0115/rpsU1	Structural component of the ribosome	2.0
BCAL0222/rplK	50S ribosomal protein L11	1.7
BCAL0224/rplJ	50S ribosomal protein L10	2.2
BCAL0225/rplL	50S ribosomal protein L7/L12	2.4
BCAL0229/rpsL	30S ribosomal protein S12	1.9
BCAL230/rpsG	30S ribosomal protein S12	1.8
BCAL0233/rpsJ	30s ribosomal protein S10	1.6
BCAL0234/rplC	50S ribosomal protein L3	2.3
BCAL0235/rplD	50S ribosomal protein L4	2.8
BCAL0236/rplW	50S ribosomal protein L23	2.5
BCAL0237/rplB	50S ribosomal protein L2	2.9
BCAL0238/rpsS	30S ribosomal protein S19	2.7
BCAL0239/rplV	50S ribosomal protein L22	2.8
BCAL0240/rpsC	30S ribosomal protein S3	2.8
BCAL0241/rplP	50S ribosomal protein L16	2.0
BCAL0242/rpmC	50S ribosomal protein L29	2.3
BCAL0243/rpsQ	30S ribosomal protein S17	2.6
BCAL0244/rplN	50S ribosomal protein L14	1.8
BCAL0245/rplX	50S ribosomal protein L24	1.5
BCAL0246/rplE	50S ribosomal protein L5	1.8
BCAL0247/rpsN	30S ribosomal protein S14	2.2
BCAL0248/rpsH	30S ribosomal protein S8	2.1
BCAL0249/rplF	50S ribosomal protein L6	1.9
BCAL0250/rplR	50S ribosomal protein L18	2.1
BCAL0251/rpsE	30S ribosomal protein S5	1.9
BCAL0252/rpmD	50S ribosomal protein L30	1.7
BCAL0253/rplO	50S ribosomal protein L15	1.9
BCAL0256/rpmJ	50S ribosomal protein L36	1.8
BCAL0257/rpsM	30S ribosomal protein S13	1.9
BCAL0258/rpsK	30S ribosomal protein S11	2.1
BCAL0259/rpsD	30S ribosomal protein S4	1.7
BCAL0261/rplQ	50S ribosomal protein L17	1.5
BCAL0799	50S ribosomal protein L25/general stress protein Ctc	1.9

BCAL0990/rpmF	50S ribosomal protein L32	1.9
BCAL1483/rpmI	50S ribosomal protein L35	2.3
BCAL1484/rpiT	50S ribosomal protein L20	2.2
BCAL1905/rpmE2	50S ribosomal protein L31 type B	2.7
BCAL1942/rplI	50S ribosomal protein L9	2.0
BCAL1943/rpsR	30S ribosomal protein S18	1.8
BCAL1945/rpsF	30S ribosomal protein S6	1.8
BCAL2715/rpmG	50S ribosomal protein L33	1.5
BCAL2765/rpsT	30S ribosomal protein S20	1.6
BCAL2928/rpsP	30S ribosomal protein S16	1.6
BCAL2950/rpsA	30S ribosomal protein S1	1.7
BCAL3347/rpsI	30S ribosomal protein S9	1.6
BCAL3348/rplM	50S ribosomal protein L13	1.7
BCAL3442/rplU	50S ribosomal protein L21	1.6
BCAL0219/tufA1	Elongation factor Tu	1.8
BCAL0231/fusA	Elongation factor G	2.5
BCAL0232/tuf	Elongation factor Tu	1.8
BCAl1012/efp2	elongation factor P	1.9
BCAL0255/infA	translation initiation factor IF-1	2.1
BCAL1507/infB	translation initiation factor IF-2	1.8
BCAL3436/proS	prolyl-tRNA synthetase	5.8
BCAL1509	tRNA pseudouridine synthase B	1.7
BCAL1508	ribosome-binding factor A	1.6
BCAL0387	GTP-dependent nucleic acid-binding protein EngD	1.9
BCAL1504	RNA pseudouridylate synthase family protein	1.6
BCAM1544	activity	2.0
BCAM1618/infA2	translation initiation factor IF-1	2.2
BCAM1700	Putative acetyltransferase similar to N-acetylases of ribosomal proteins	2.0
BCALr00219	tRNA-Trp	1.8
BCALr0164	tRNA-Ala	2.0
BCALr0217b	tRNA-Ile	1.8
BCALr0218a	tRNA-Tyr	1.9
BCALr0218b	tRNA-Gly	1.6
BCALr0218c	tRNA-Thr	7.1

BCALr0409c	tRNA-Ala	2.3
BCALr0472	tRNA-Phe	2.7
BCALr0970a	tRNA-Asn	5.3
BCALr1551a	tRNA-Leu	2.9
BCALr1614	tRNA-Met	5.7
BCALr1993b	tRNA-Val	32.6
BCALr2006	tRNA-Leu	1.8
BCALr2125c	tRNA-Asp	2.1
BCALr2125e	tRNA-Asp	1.7
BCALr2145	tRNA-Ser	5.4
BCALr2219	tRNA-Met	4.4
BCALr2236	tRNA-Val	32.7
BCALr2852a	tRNA-Cys	1.6
BCALr2852d	tRNA-Gly	1.6
BCALr3009b	tRNA-Arg	1.8
BCALr3016	tRNA-Ser	1.9
BCALr3205	tRNA-Lys	5.7
BCALr3443	tRNA-Pro	2.4
BCAMr0918	tRNA-Met	1.8
BCAMr1023	tRNA-Sec	3.9
Motility and adherence		
BCAL0140/flhB	Flagellar biosynthesis protein FlhB	2.0
BCAL0142/flhF	Flagellar biosynthesis regulator FlhF	1.7
BCAL0143	Putative Flagellar biosynthesis protein	2.1
BCAL0520/fliK	Putative Flagellar hook-length control protein FliK	1.8
BCAL0521/fliJ	Flagellar biosynthesis chaperone; involved in flagella export	1.7
BCAL0524/fliG	Flagellar motor switch protein G	1.5
BCAL0564/flgB	Flagellar basal body rod protein FlgB	1.7
BCAL0566/flgD	Flagellar basal body rod modification protein	1.8
BCAL0567/flgE	Flagellar hook protein FlgE	1.8
BCAL0568/flgF	Flagellar basal body rod protein FlgF	1.9
BCAL0569/flgG	Flagellar basal body rod protein FlgG	1.8

BCAL0570/flgH	Flagellar basal body L-ring protein	1.6
BCAL0571/flgI	Flagellar basal body P-ring protein	1.7
BCAL3505/fliN	Flagellar motor switch protein FliN	1.8
BCAL0140/flhB	Putative Flagellar protein FhlB	2.41
Protein folding and modification		
BCAL1997	trigger factor; similar to proteins with peptidyl-prolyl cis-trans isomerase activity	1.5
BCAL2847	Putative methionine aminopeptidase	1.6
BCAL1025	Putative methionine aminopeptidase	1.7
BCAL1703	metallo peptidase, subfamily M20D	2.4
BCAS0397	metallo peptidase, subfamily M20D	3.2
BCAM0628	Putative low molecular weight phosphotyrosine protein phosphatase	4.4
BCAS0065	Putative glutathione S-transferase	1.7
BCAL1876/hflC	Component of the HflK-HflC complex which is located in the outer membrane-bounded periplasmic space and that is involved in the regulation of the FstH protease complex	1.7
BCAL1877/hflK	Component of the HflK-HflC complex which is located in the outer membrane-bounded periplasmic space and that is involved in the regulation of the FstH protease complex	1.7
Ion homeostasis		
Iron uptake		
BCAL1697/orbJ	ornibactin biosynthesis non-ribosomal peptide synthase	1.9
BCAL1698/orbK	ornibactin biosynthesis protein	3.0
BCAL1699/pvdA	L-ornithine N(5)-oxygenase required for iron assimilation	4.9
BCAL1700/orbA	ornibactin receptor precursor	5.9
BCAL1701/pvdF	ornibactin synthetase F	4.1
BCAL1702/orbL	Putative ornibactin biosynthesis protein	3.1
BCAL1371	Putative TonB-dependent siderophore receptor	1.6
BCAM0564	Putative TonB-dependent siderophore receptor	1.5
BCAM1571	Putative TonB-dependent siderophore receptor	1.9
BCAM2626/huvA	TonB-dependent hemoglobin/transferrin/lactoferrin receptor; heme receptor	2.0
BCAM2627/hmuS	Putative heme degradation protein	1.7
BCAL0727	Putative magnesium chelatase subunit	1.6
BCAL1622/mopII	molybdenum-pterin binding protein II	2.2
BCAL2102	Probable glutaredoxin presumed to be involved in arsenate reduction	1.6

BCAM0447	Putative exported multicopper oxidase presumed to be involved in copper transport	1.7
BCAM0539	Similar to rhodanese-related sulfurtransferases	1.7

Replication, transcription and transcriptional regulation

Transcriptional regulators

BCAL0144/sigK	Sigma factor 70 SigK	1.6
BCAL0185	Transcriptional regulator of the LysR family	1.6
BCAL0602	Transcriptional regulator of the MerR family	1.7
BCAL0777	Transcriptional regulator of the GntR family	2.5
BCAl0914	Putative DNA binding protein of the HipB family of regulators	1.9
BCAL1503	Putative transcriptional regulator protein	1.6
BCAL2048	Transcriptional regulator of the GntR family	1.7
BCAL2732	Transcriptional regulator similar to CspC which is involved in the control of stress response in E. coli	2.6
BCAL3363	Transcriptional regulator of the TetR family	2.0
BCAM0222	Putative DNA-binding protein	4.1
BCAM0240/cciR	N-acylhomoserine lactone dependent regulatory protein	1.7
BCAM0514	Transcriptional regulator of the TetR family; similar to the E. coli multidrug efflux regulator AcrR	1.7
BCAM0552	Putative sigma-54 dependent transcriptional regulator	2.2
BCAM0731	Transcriptional regulator of the MarR family	1.5
BCAM0958	Transcriptional regulator similar to the ArsR from E. coli which is involved in arsenic resistance	1.6
BCAM0977	Transcriptional regulator of the AsnC family	1.8
BCAM1184	Putative transcriptional regulator of the GntR family; similar to Aro8 which is involved in the control of catabolism of aromatic aminoacids	1.6
BCAM1305	Putative transcriptional regulator	1.6
BCAM1437	Transcriptional regulator of the MarR family	1.5
BCAM1619	Putative DNA-binding cold-shock protein; similar to CspD	6.9
BCAM1719	Transcriptional regulator of the LysR family	1.5
BCAM1975	Transcriptional regulator of the AraC family	2.3
BCAM1981	Putative transcriptional regulator of the LuxR Superfamily	2.0
BCAM2589	Putative transcriptional repressor of IciR family	1.7

BCAS0115	Transcriptional regulator of the GntR family	1.7
BCAS0225/shvR	shiny variant regulator, ShvR	1.5
BCAS0376	Transcriptional repressor of the GntR family	1.8
BCAS0380	Transcriptional repressor of the TetR family	1.6
BCAS0382	Transcriptional regulator of the AnsC family	1.9
BCAS0393	Transcriptional regulator of the AraC family	3.3
Bcen_1449	Transcriptional regulator of the LysR family	3.5
BCAL1557	Transcriptional regulator of the GntR family; similar to FadR which is involved in the regulation of fatty acid biosynthesis	2.0
Transcription		
BCAL0368/cspD	Similar to cold shock protein CspD from E. coli which is a predicted RNA chaperone	14.2
BCAL0226/rpoB	DNA-directed RNA polymerase subunit beta	1.8
BCAL0227/rpoC	DNA-directed RNA polymerase subunit beta'	1.7
BCAL0260/rpoA	DNA-directed RNA polymerase subunit alpha	1.6
BCAL1901/rho	transcription termination factor rho	1.6
BCAL2117	Putative ATP-dependent RNA helicase	1.8
BCAL2348/pnp	polynucleotide phosphorylase/polyadenylase	2.6
Replication		
BCAM1810	Putative cold shock protein presumed to be involved in negative regulation of DNA replication	6.9
BCAL1944	Putative primosomal replication protein	4.7
Others		
BCAL0154	Histone-like nucleoid-structuring (H-NS) protein	1.8
BCAL2295	Putative chromosome condensation protein	2.0
BCALr0914	RNA molecule of unknown function	2.1
BCALr3349	RNA molecule of unknown function	1.5
BCAL2888/rne1	ribonuclease E 1	2.5
BCAS0492	Putative endoribonuclease of the L-psp family protein	2.2
BCAL2931	radical SAM superfamily protein; similar to photolyases involved in DNA repair	1.7

Protein trafficking and secretion		
BCAL0254/secY	Preprotein translocase subunit SecY	2.1
BCAL0325/tatC	Sec-independent protein translocase protein TatC	1.6
BCAL0426	Putative inner membrane protein translocase component YidC	1.7
Signalling		
BCAM0645	Two-component regulatory system, sensor kinase protein	1.6
BCAM1705	two-component regulatory system, sensor kinase protein	2.4
Stress-response		
BCAL1437	Putative oxidoreductase	1.5
BCAL1766	Peroxiredoxin	1.6
BCAL3418	Putative thioredoxin protein	1.7
BCAM0181	Putative catalase	4.5
BCAS0437	Predicted peroxiredoxin	2.2
Phage-related genes		
BCAL1317	Putative phage integrase	1.6
BCAL1519	Putative transposase	1.8
BCAS0553/BcepMu2	Mu-like prophage protein gp16	1.9
Unknown		
BCAL0146		1.5
BCAL0425		1.7
BCAL0440		1.9
BCAL0506		1.6
BCAL0594		1.9
BCAl0683		2.1
BCAL0765		2.7
BCAL0768		1.6
BCAL0784		2.2
BCAL1030	Similar to pirin	1.7
BCAL1085		1.5

BCAL1105	1.9
BCAL1378	2.0
BCAL1500	1.7
BCAl1742	1.5
BCAL1756	1.5
BCAL1765	2.5
BCAL1798	1.5
BCAL1875	1.8
BCAL1878	1.9
BCAL2054	2.0
BCAL2055	1.5
BCAL2201	2.0
BCAL2286	1.8
BCAL2451	1.7
BCAL2554	1.8
BCAL2608	1.6
BCAL2734	1.5
BCAL2905	1.7
BCAL2956	6.3
BCAL3283	2.6
BCAM0243	1.8
BCAM0542	1.8
BCAM0629	1.5
BCAM0693	1.6
BCAM0705	1.5
BCAM0841	1.7
BCAM0895	1.6
BCAM0902	1.6
BCAM1077	1.7
BCAM1215	1.7
BCAM1220	1.9
BCAM1279	1.8
BCAM1390	1.8
BCAM1425	1.5

BCAM1677	1.8
BCAM1811	2.0
BCAM1987	1.8
BCAM1991	1.5
BCAM2002	1.6
BCAM2527	1.8
BCAM2647	5.4
BCAM2676	1.7
BCAM2685	1.6
BCAM2717	1.9
BCAS0137	1.5
BCAS0290	1.7
BCAS0296	1.6
BCAS0315	1.9
BCAS0344	1.5
BCAS0394	3.0
BCAS0413	1.8
BCAS0414	3.0
BCAS0463	1.9
BCAS0596	1.9
BCAS0750	2.1
BCAS0755	1.6
pBCA015	2.0
BCAL1414	1.6
BCAM0608	1.6
BCAM1338	2.5
BCAM1562	2.0
BCAM1814	2.3
BCAM2530	1.5
BCAM2598	2.2
BCAL0518	1.7
BCAL3362	1.6
BCAS0392	2.4
BCAS0395	2.6

BCAS0396	2.9
BCAL2619	2.1
BCAL2811	1.7
BCAM0034	2.9
BCAM1140	1.6
BCAM2648	1.9
BCAS0046	1.5
BCAS0385	3.2
BCAS0278	1.7
BCAS0386	4.0
BCAS0445	1.9
BCAM1147	1.6
BCAM0035	2.2
BCAM1221	1.6
BCAM2570	2.1
BCAS0384	2.6
BCAM0039	6.0
BCAM2298	1.9
BCAS0138	1.7
BCAM1960	1.7
BCAL3435	1.8
Bcen_2828	2.0

Genes up-regulated in IST4113, compared to IST439, sorted by gene number

pBCA015
Bcen_2828
Bcen_1449
Bcen_1448
BCAS0755
BCAS0750
BCAS0627
BCAS0625
BCAS0596
BCAS0575
BCAS0574
BCAS0553
BCAS0497
BCAS0496
BCAS0495
BCAS0494
BCAS0492
BCAS0463
BCAS0445
BCAS0438
BCAS0437
BCAS0426
BCAS0425
BCAS0420
BCAS0419
BCAS0417
BCAS0414
BCAS0413
BCAS0397
BCAS0396
BCAS0395
BCAS0394

BCAS0393
BCAS0392
BCAS0391
BCAS0390
BCAS0389
BCAS0388
BCAS0386
BCAS0385
BCAS0384
BCAS0383
BCAS0382
BCAS0381
BCAS0380
BCAS0376
BCAS0346
BCAS0344
BCAS0343
BCAS0342
BCAS0341
BCAS0340
BCAS0315
BCAS0305
BCAS0296
BCAS0290
BCAS0278
BCAS0225
BCAS0211
BCAS0138
BCAS0137
BCAS0136
BCAS0122
BCAS0115
BCAS0114
BCAS0113

BCAS0110
BCAS0066
BCAS0065
BCAS0054
BCAS0053
BCAS0046
BCAMr1023
BCAMr0918
BCAM2806
BCAM2783
BCAM2726
BCAM2725
BCAM2724
BCAM2717
BCAM2697
BCAM2685
BCAM2676
BCAM2675
BCAM2674
BCAM2648
BCAM2647
BCAM2627
BCAM2626
BCAM2620
BCAM2618
BCAM2598
BCAM2589
BCAM2570
BCAM2545
BCAM2530
BCAM2529
BCAM2527
BCAM2526
BCAM2509

BCAM2316
BCAM2304
BCAM2302A
BCAM2301
BCAM2300
BCAM2299
BCAM2298
BCAM2251
BCAM2250
BCAM2249
BCAM2248
BCAM2247
BCAM2188
BCAM2122
BCAM2002
BCAM1991
BCAM1988
BCAM1987
BCAM1986
BCAM1981
BCAM1979
BCAM1978
BCAM1975
BCAM1974
BCAM1961
BCAM1960
BCAM1814
BCAM1811
BCAM1810
BCAM1774
BCAM1773
BCAM1772
BCAM1771
BCAM1770

BCAM1761
BCAM1719
BCAM1705
BCAM1704
BCAM1700
BCAM1678
BCAM1677
BCAM1676
BCAM1619
BCAM1618
BCAM1588
BCAM1582
BCAM1571
BCAM1566
BCAM1565
BCAM1564
BCAM1563
BCAM1562
BCAM1560
BCAM1544
BCAM1455
BCAM1449A
BCAM1437
BCAM1427
BCAM1425
BCAM1391
BCAM1390
BCAM1389
BCAM1387
BCAM1386
BCAM1379
BCAM1378
BCAM1377
BCAM1376

BCAM1338
BCAM1315
BCAM1305
BCAM1286
BCAM1279
BCAM1221
BCAM1220
BCAM1219
BCAM1215
BCAM1184
BCAM1151
BCAM1147
BCAM1140
BCAM1126
BCAM1125
BCAM1124
BCAM1123
BCAM1122
BCAM1121
BCAM1077
BCAM0978
BCAM0977
BCAM0976
BCAM0958
BCAM0902
BCAM0895
BCAM0841
BCAM0811
BCAM0810
BCAM0746
BCAM0731
BCAM0712
BCAM0705
BCAM0693

BCAM0692
BCAM0645
BCAM0629
BCAM0628
BCAM0612
BCAM0611
BCAM0610
BCAM0608
BCAM0606
BCAM0564
BCAM0563
BCAM0561
BCAM0560
BCAM0558
BCAM0557
BCAM0556
BCAM0555
BCAM0552
BCAM0551
BCAM0550
BCAM0543
BCAM0542
BCAM0540
BCAM0539
BCAM0514
BCAM0447
BCAM0441
BCAM0439
BCAM0382
BCAM0243
BCAM0240
BCAM0238
BCAM0223
BCAM0222

BCAM0220
BCAM0219
BCAM0214
BCAM0207
BCAM0201
BCAM0185
BCAM0184
BCAM0182
BCAM0181
BCAM0180
BCAM0171
BCAM0166
BCAM0153
BCAM0121
BCAM0098
BCAM0039
BCAM0036
BCAM0035
BCAM0034
BCALr3443
BCALr3349
BCALr3205
BCALr3016
BCALr3009b
BCALr2852d
BCALr2852a
BCALr2236
BCALr2219
BCALr2145
BCALr2125e
BCALr2125c
BCALr2006
BCALr1993b
BCALr1614

BCALr1551a
BCALr0970a
BCALr0914
BCALr0472
BCALr0409c
BCALr0218c
BCALr0218b
BCALr0218a
BCALr0217b
BCALr0164
BCALr00219
BCAL3505
BCAL3442
BCAL3438
BCAL3436
BCAL3435
BCAL3418
BCAL3363
BCAL3362
BCAL3348
BCAL3347
BCAL3285
BCAL3283
BCAL3282
BCAL3138
BCAL3051
BCAL3050
BCAL3049
BCAL2956
BCAL2950
BCAL2931
BCAL2928
BCAL2905
BCAL2888

BCAL2867
BCAL2847
BCAL2822
BCAL2811
BCAL2765
BCAL2759
BCAL2734
BCAL2732
BCAL2729
BCAL2725
BCAL2718
BCAL2717
BCAL2715
BCAL2619
BCAL2608
BCAL2554
BCAL2466
BCAL2451
BCAL2354
BCAL2348
BCAL230
BCAL2295
BCAL2286
BCAL2244
BCAL2241
BCAL2240
BCAL2224
BCAL2201
BCAL2180
BCAL2166
BCAL2146
BCAL2122
BCAL2117
BCAL2102

BCAL2063
BCAL2061
BCAL2055
BCAL2054
BCAL2053
BCAL2052
BCAL2051
BCAL2050
BCAL2049
BCAL2048
BCAL1997
BCAL1945
BCAL1944
BCAL1943
BCAL1942
BCAL1926
BCAL1913
BCAL1911
BCAL1910
BCAL1909
BCAL1908
BCAL1905
BCAL1901
BCAL1887
BCAL1878
BCAL1877
BCAL1876
BCAL1875
BCAL1872
BCAL1862
BCAL1798
BCAL1796
BCAL1775
BCAL1766

BCAL1765
BCAL1756
BCAl1742
BCAL1703
BCAL1702
BCAL1701
BCAL1700
BCAL1699
BCAL1698
BCAL1697
BCAL1671
BCAL1670
BCAL1669
BCAL1668
BCAL1622
BCAL1621
BCAL1620
BCAL1619
BCAL1557
BCAL1519
BCAL1509
BCAL1508
BCAL1507
BCAL1504
BCAL1503
BCAL1500
BCAL1484
BCAL1483
BCAL1437
BCAL1436
BCAL1435
BCAL1431
BCAL1423
BCAL1414

BCAL1413A
BCAL1378
BCAL1377
BCAL1371
BCAL1317
BCAL1252
BCAL1225
BCAL1223
BCAL1105
BCAL1092
BCAL1085
BCAL1081
BCAL1080
BCAL1079
BCAL1030
BCAL1025
BCAl1012
BCAL0990
BCAL0915
BCAl0914
BCAL0906
BCAL0799
BCAL0784
BCAL0781
BCAL0780
BCAL0779
BCAL0778
BCAL0777
BCAL0768
BCAL0766
BCAL0765
BCAL0727
BCAL0713
BCAL0712

BCAL0711
BCAl0683
BCAL0640
BCAL0603
BCAL0602
BCAL0600
BCAL0599
BCAL0598
BCAL0596
BCAL0595
BCAL0594
BCAL0571
BCAL0570
BCAL0569
BCAL0568
BCAL0567
BCAL0566
BCAL0564
BCAL0524
BCAL0521
BCAL0520
BCAL0518
BCAL0506
BCAL0482
BCAL0440
BCAL0426
BCAL0425
BCAL0391
BCAL0387
BCAL0368
BCAL0325
BCAL0261
BCAL0260
BCAL0259

BCAL0258
BCAL0257
BCAL0256
BCAL0255
BCAL0254
BCAL0253
BCAL0252
BCAL0251
BCAL0250
BCAL0249
BCAL0248
BCAL0247
BCAL0246
BCAL0245
BCAL0244
BCAL0243
BCAL0242
BCAL0241
BCAL0240
BCAL0239
BCAL0238
BCAL0237
BCAL0236
BCAL0235
BCAL0234
BCAL0233
BCAL0232
BCAL0231
BCAL0229
BCAL0227
BCAL0226
BCAL0225
BCAL0224
BCAL0222

BCAL0219
BCAL0206
BCAL0204
BCAL0185
BCAL0159
BCAL0154
BCAL0151
BCAL0146
BCAL0144
BCAL0143
BCAL0142
BCAL0140
BCAL0140
BCAL0115
BCAL0110
BCAL0075
BCAL0074
BCAL0073
BCAL0055
BCAL0037
BCAL0030
BCAL0019
