# Supplementary material for: Genomic Expression Analysis Reveals Strategies of Burkholderia cenocepacia to Adapt to Cystic Fibrosis Patients' Airways and Antimicrobial Therapy
Source: PLoS One. 2011 Dec 21;6(12):e28831. doi: 10.1371/journal.pone.0028831 (PMC3244429; doi:10.1371/journal.pone.0028831)
Supplement: Table S3 — Full list of genes down-regulated (above 1.5-fold) in B. cenocepacia IST4113, compared to IST439. The genes whose transcript level in the B. cenocepacia IST4113 variant were at least 1.5-fold lower than the levels attained in IST439 were selected. The description of gene function is based on the information available in the “Burkholderia Genome Database” and in “KEGG Pathways” database and the functional clustering was performed using the functional classes shown in Figure 1. Genes specific of the AU1054 strain are shaded in grey. (PDF) [file pone.0028831.s003.pdf]

Table S2 - List of genes down-regulated (above 1.5-fold) in *B. cenocepacia* IST4113, compared to IST439. The genes whose transcript level in the B. cenocepacia IST4113 variant were at least 1.5-fold lower than the levels attained in IST439 were selected. The description of gene function is based on the information available in the "Burkholderia Genome Database" and in "KEEG pathways" database and the functional clustering was performed using the functional classes shown in Figure 1. Genes specific of the AU1054 strain are shaded in grey

Gene/ORF	Function	mRNA IST4113 / mRNA IST439
Carbohydrate metabolism		
Electron transport and oxidative phosphorylation		
BCAL0328/petA	Ubiquinol-cytochrome c reductase iron-sulfur subunit; involved in aerobic respiration	-1.9
BCAL0329/petB	Cytochrome b of the cytochrome *bc* complex; involved in aerobic respiration	-2.4
BCAL0330/petC	Cytochrome c1 precursor	-1.6
BCAL0388	Ubiquinone biosynthesis hydroxylase family protein	-2.4
BCAL0784/cydA	Cytochrome d ubiquinol oxidase subunit II (cytochrome *bd*)	-1.6
BCAL0785/cydB	Cytochrome d ubiquinol oxidase subunit II (cytochrome bd)	-2.3
BCAL2141/cyoD	Cytochrome O ubiquinol oxidase protein	-2.2
BCAL2142/cyoC	Cytochrome o ubiquinol oxidase subunit III	-2.1
BCAL2143/cyoB	Ubiquinol oxidase polypeptide I	-2.6
BCAL2144/cyoA	Ubiquinol oxidase polypeptide II precursor	-2.3
BCAL2472	Alpha,alpha-trehalose-phosphate synthase	-1.9
BCAL2680	Uroporphyrin-III C-methyltransferase; involved in heme biosynthesis	-1.5
BCAL2899	4Fe-4S ferredoxin	-1.7
BCAL3296	4-hydroxybenzoate octaprenyltransferase involved in ubiquinone biosynthesis	-1.9
BCAM0946	Putative cytochrome b561	-2.0
BCAM1734	Predicted gluconate dehydrogenase	-1.6
BCAM2319	Iron-sulphur Rieske protein	-1.7
BCAM2591	Putative cytochrome P450 iron-sulfur protein	-2.2

Krebs cycle		
BCAL2746	Putative citrate synthase	-1.9
BCAM0962	2-methylcitrate dehydratase	-1.5
BCAM0964	Putative lyase involved that converts citrate into oxaloacetate	-2.0
BCAM0965/mdh	Malate dehydrogenase	-1.9
BCAM1833/acnB	Bifunctional aconitate hydratase 2/2-methylisocitrate dehydratase	-2.8
BCAS0023	Aldolase/citrate lyase family protein predicted to be involved in conversion of citrate into oxaloacetate	-1.6
BCAM2607	Putative NADH:flavin oxidoreductase/NADH oxidase	-1.6
Phenylacetate degradation		
BCAL0212/paaD	Phenylacetic acid degradation protein PaaD	-1.7
BCAL0213/paaC	Phenylacetic acid degradation protein PaaC	-1.6
BCAL0404/paaK	Phenylacetate-coenzyme A ligase	-2.2
BCAL0405/paaI	Phenylacetic acid degradation protein	-2.2
BCAL0406/paaG	Enoyl-CoA hydratase involved in phenylacetate degradation	-2.0
BCAL0407/paaF	Beta-ketoadipyl CoA thiolase involved in phenylacetate degradation	-1.9
BCAL0408/paaZ	Putative phenylacetic acid degradation oxidoreductase	-2.4
BCAM2568	Beta-ketoadipyl CoA thiolase; involved in phenylacetate degradation	-1.8
Biosynthesis of vitamines and coenzymes		
BCAL0503	Involved in biosynthesis of vitamine B12	-1.6
BCAL1047/pdxY	Pyridoxamine kinase involved in biosynthesis of vitamine B12	-1.6
BCAL3276/ppnK	NAD(+)/NADH kinase family protein	-1.7
BCAM0138	Phosphoglycerate mutase family protein; predicted to be involved in vitamine B12 biosynthesis	-1.6
BCAM2080	Bifunctional nicotinamide mononucleotide adenylyltransferase/ADP-ribose pyrophosphatase involved in NAD^+ biosynthesis/degradation	-1.7
BCAS0253	2-dehydropantoate 2-reductase involved in pantothenate biosynthesis	-1.7
Metabolism of ketone bodies		
BCAL1472/scoA	Succinyl-CoA:3-ketoacid-coenzyme A transferase subunit A; involved in synthesis and degradation of ketone bodies	-1.6

BCAL1473/scoB	Succinyl-CoA:3-ketoacid-coenzyme A transferase subunit B; involved in synthesis and degradation of ketone bodies	-1.6
BCAM0023/adc	Acetoacetate decarboxylase involved in degradation of ketone bodies	-1.6
Others		
BCAM2791	Feruloyl-CoA synthase involved in ferulic acid metabolism	-2.0
BCAL0064	Acetaldeyde dehydrogenase	-1.7
BCAL0476	HpcH/HpaI aldolase/citrate lyase family protein involved in biosynthesis of tartronate semialdeyde	-1.6
BCAL0536/fpr	Ferredoxin-NADP reductase	-1.6
BCAL1390/bcsZ	Endo-1,4-D-glucanase	-1.8
BCAL2041/hyuE	Hydantoin racemase	-1.9
BCAL2206	Phasin-like protein; predicted to be involved in metabolism of PHAs	-3.0
BCAL3379	Ureidoglycolate hydrolase	-2.1
BCAL3380	Allantoicase	-1.9
BCAM1570	Alcohol dehydrogenase	-8.3
BCAM2343/betA	Choline dehydrogenase	-1.5
BCAM2710	Putative acetyl-CoA synthetase involved in formation of acetyl-CoA from acetate	-2.5
BCAS0034	Metallo-beta-lactamase superfamily protein	-1.8
BCAM1430	Metallo-beta-lactamase superfamily protein	-1.6
BCAS0320	Isoquinoline 1-oxidoreductase alpha subunit	-2.3
BCAM1734	Predicted gluconate dehydrogenase	-1.6
**Aminoacid and nitrogen metabolism**		
Histidine biosynthesis		
BCAL0317/hisA	1-(5-phosphoribosyl)-5-[(5- phosphoribosylamino)methylideneamino] imidazole-4-carboxamide isomerase; involved in histidine biosynthesis	-1.8
BCAL0319/hisI	Phosphoribosyl-AMP cyclohydrolase	-1.6
Polyamine biosynthesis		
BCAM0416/Agmat	Putative agmatinase involved in spermidine biosynthesis	-1.6
BCAM0592	Putative orthinine cyclodeaminase involved in proline biosynthesis from ornithine	-1.5

BCAM1111	Ornithine decarboxylase involved in conversion of ornithine into putrescine	-1.8
BCAM1112	Arginine decarboxylase involved in conversion of arginine into agmatine; involved in polyamine biosynthesis	-1.5
BCAL3390	Spermidine synthase	-1.6
Leucine, valine and alanine metabolism		
BCAL0068	Thiamine pyrophosphate protein; involved in leucine and valine biosynthesis	-1.7
BCAM2430	Putative biotin carboxylase involved in valine/leucine/isoleucine degradation	-1.7
BCAM2431	Enoyl-CoA hydratase; involved in valine/leucine/isoleucine degradation	-1.5
BCAM2432	Putative biotin-dependent carboxyl transferase carboxylase involved in valine/leucine/isoleucine degradation	-1.7
BCAM2433	Putative acyl-CoA dehydrogenase; involved in valine/leucine/isoleucine degradation	-1.6
Tryptophan metabolism		
BCAL0398/trpG	Anthranilate synthase component II; involved in tryptophan biosynthesis	-1.5
BCAL2441	Chorismate lyase; involved in biosynthesis of aromatic aminoacids	-2.1
BCAL2791	Putative kynureninase involved in tryptophan catabolism	-1.8
BCAL2792	Putative tryptophan 2,3-dioxygenase	-1.7
Others		
BCAL0460	into 3-phosphohidroxypyruvate in the biosynthesis of serine	-1.5
BCAL2352	Putative carbonic anhydrase	-1.5
BCAL3107/ureE	Urease accessory protein; involved in urea catabolism	-1.6
BCAL3359	Putative glutamate dehydrogenase	-2.2
BCAL0042/putA	Proline dehydrogenase/pyrroline-5-carboxylate dehydrogenase	-1.9
BCAM1353/ald	Alanine dehydrogenase involved in conversion of alanine into pyruvate	-2.3
BCAM2176/gsa	Glutaminase involved in conversion of glutamine into glutamate	-1.5
BCAM2326/glyA	Serine hydroxymethyltransferase	-2.0
BCAM2392/sdaB	Putative L-serine dehydratase involved in conversion of pyruvate into serine	-1.5
BCAM2325	Putative dipeptidase	-1.9

**Purine and pyrimidine metabolism**		
BCAL1975	Putative diguanylate cyclase	-1.6
BCAL3428	Ribonucleotide-diphosphate reductase subunit beta involved in conversion of GDP to dGDP	-1.9
BCAM2006	Aspartate carbamoyltransferase involved in pyrimidine biosynthesis	-1.9
BCAM2822	Putative diguanylate cyclase; involved in c-diGMP biosynthesis	-1.6
Lipid metabolism		
BCAL0046	Putative fatty-acid CoA ligase	-1.5
BCAL0047	Putative acyl-CoA dehydrogenase	-1.8
BCAL1046/plcN	Putative non-hemolytic phospholipase C	-1.7
BCAM0408	Putative phospholipase C	-2.2
BCAL1247	Similar to phosphatidylserine/phosphatidylglycerophosphate/cardiolipin synthases	-2.2
BCAL1249	Putative poly-beta-hydroxyalkanoate depolymerase depolymerase	-3.1
BCAL2690	Short chain dehydrogenase; predicted to have 3-oxoacyl-[acyl-carrier-protein] reductase activity	-2.0
BCAL2783	Putative cyclopropane-fatty-acyl-phospholipid synthase	-2.1
BCAL3191	Putative glutaryl-CoA dehydrogenase	-1.9
BCAM0254	Putative acyl carrier protein	-1.5
BCAM0774	Poly[D-(-)-3-hydroxybutyrate] depolymerase	-1.6
BCAM2340/phaG	Putative (R)-3-hydroxydecanoyl-ACP:CoA transacylase	-1.8
Sulphur metabolism		
BCAL2681/cysN	Putative sulfate adenylyltransferase subunit 1	-1.6
BCAL2682/cysD1	Sulfate adenylyltransferase subunit 2	-1.8
BCAL2683/cysH	Phosphoadenosine phosphosulfate reductase	-1.5
Transport		
Sugars		
BCAL0282	Periplasmic component of an ABC transporter presumed to be involved in glycerol-3-phosphate transport	-1.8
BCAL0283	Permease component of an ABC transporter presumed to be involved in glycerol-3-phosphate transport	-1.8

BCAL0543	Major Facilitator Superfamily protein similar to sugar efflux permeases	-1.8
BCAL3381/dctA	C4-dicarboxylate transporter DctA	-2.3
BCAM0086	Putative sodium:dicarboxylate symporter family protein	-1.5
BCAM1361	Permease component of an ABC transporter presumed to be involved in sugar transport	-1.6
BCAM2590	Major Facilitator Superfamily protein; similar to sugar transporters	-1.8
BCAS0017	Putative fusaric acid resistance transporter protein	-1.6
BCAS0230	ATPase component of a Putative sugar ABC transporter	-1.6
BCAS0259	Putative sodium:dicarboxylate symporter family protein	-1.7
Phosphate		
BCAL1271/pstC	Phosphate transporter permease subunit PstC	-1.9
BCAL1272/pstA	Phosphate transporter permease subunit PtsA	-2.4
BCAL1273/pstB	ATPase component of a Putative phosphate ABC transporter	-2.5
Drugs		
BCAL1510	Subunit of a MFS transporter predicted to be involved in drug efflux	-2.3
BCAL1511/emrA	Subunit of a MFS transporter predicted to be involved in drug efflux	-1.7
BCAM2337	Putative multidrug resistance transporter protein	-1.8
Ions		
BCAL1400	Sodium/hydrogen exchanger family protein	-1.5
BCAL2305	Putative potassium channel subunit	-2.4
BCAM1745	Putative magnesium-transporting ATPase	-1.7
Aminoacids and polyamines		
BCAM0096	ATPase component of a Putative polyamine ABC transporter	-2.0
BCAM0952	ATPase component of a Putative polyamine ABC transporter	-1.6
BCAM0954	Permease component of an ABC transporter presumed to be involved in polyamine transport	-1.5
BCAM1477/aroP	Aromatic amino acid transport protein	-1.8
BCAM2469	Putative amino-acid permease	-1.5
BCAS0240	Periplasmic component of a Putative aminoacid ABC transporter	-1.6

Others		
BCAL0120	Aquaporin Z	-1.5
BCAL1418	Major Facilitator Superfamily protein	-1.8
BCAL1647	Nodulation ABC transporter NodI	-1.6
BCAL2814	ATPase component of a Putative ABC transporter	-1.5
BCAM0584	Major Facilitator Superfamily protein	-1.8
BCAM2208	Putative permease protein	-1.5
BCAM2381	Periplasmic component of a Putative ABC transporter involved in choline efflux	-1.7
BCAM2383	ATPase component of a Putative ABC transporter	-1.5
BCAM2556	Putative purine nucleoside permease	-2.7
Cell envelope and outer membrane		
BCAL0067	ADP-heptose:LPS heptosyltransferase	-1.6
BCAL3110/waaA	3-deoxy-D-manno-octulosonic-acid transferase involved in biosynthesis of O-antigen	-1.6
BCAL3111/wbxY	Protein of unknown function that is involved in biosynthesis of O-antigen	-1.6
BCAL1076	Putative outer membrane protein of unknown function; similar to OmpC	-1.7
BCAL1829	Putative outer membrane protein	-5.3
BCAL1956	Putative lipoprotein	-2.0
BCAL2024/mscl	Large-conductance mechanosensitive channel	-2.0
BCAL2205	D-alanyl-D-alanine endopeptidase (penicillin-binding protein precursor)	-1.6
BCAL2645	Putative OmpA family membrane protein; similar to P. aeruginosa OprF	-2.6
BCAL2829/htrA	HtrA protease; serine-type endopeptidase	-1.7
BCAL2958/ompA	Putative ompA family protein	-1.8
BCAL3008	Putative outer membrane porin protein; similar to P. aeruginosa OprE	-5.2
BCAL3166	Putative lipoprotein containing a Putative phospholipid-binding domain	-1.5
BCAL3473	Putative outer membrane porin	-1.6
BCAM0186/bclA	Lectin	-2.1
BCAS0292	Putative lectin	-1.7
BCAM0224	Putative haemagglutinin-related autotransporter protein	-1.6
BCAM0384	Putative lipoprotein	-2.5
BCAM505	Putative membrane-attached protein	-2.1
BCAM1015	Putative porin	-2.7
BCAM1550	Putative peptidoglycan-associated lipoprotein	-2.3

BCAM1598	Putative lipoprotein	-1.7
BCAM1737	Putative exported glycoprotein	-1.6
BCAM1738	Putative penicillin-binding protein	-1.8
BCAM1740	Putative lipoprotein	-1.5
BCAM1787	Putative outer membrane porin; 30% similarity with the probable P. aeruginosa outer membrane receptor for iron transport PA4514	-1.7
BCAM1931	Putative outer membrane porin	-3.4
BCAM2067/uppS	Putative undecaprenyl pyrophosphate synthetase involved in peptidoglycan biosynthesis	-3.7
BCAM2143/adhA	Cable pilus associated adhesin protein	-2.0
BCAM2169	Putative outer membrane autotransporter	-2.4
BCAM2307/zmpB	Zinc metalloprotease ZmpB	-3.5
BCAS0236	Putative haemagglutinin-related autotransporter protein	-2.3
BCAS0293/aidA	Nematocidal protease AidA	-9.1
BCAS0409/zmpA	Zinc metalloprotease ZmpA	-7.8
BCAS0460	Putative porin protein	-2.1
Protein synthesis		
BCAL0024/gidA	tRNA uridine 5-carboxymethylaminomethyl modification enzyme	-1.6
BCALr1625	tRNA-His	-1.5
Motility and adherence		
BCAL0124/flhD	Transcriptional activator of flagellar biosynthesis	-1.6
BCAL0126/motA	Flagellar motor protein MotA	-2.3
BCAL0127/motB	Flagellar motor protein MotB	-3.3
BCAL0128/cheY	Chemotaxis two-component response regulator CheY	-2.1
BCAL0131/tar	Methyl-accepting chemotaxis protein	-2.2
BCAL1677	Putative type-1 fimbrial protein	-1.5
BCAL2636	Putative fimbriae chaperone	-1.9
BCAS0181	Putative methyl-accepting chemotaxis protein that functions in the transfer of receptor signals to the flagella	-1.8
BCAL0480	Rod shape-determining protein MreD	-4.1
BCAL0136	Chemotaxis regulator CheZ	-2.7

Protein folding and modification		
BCAL0540	Putative ATPase similar to Lon proteases	-1.8
BCAL1233	Putative heat shock Hsp20-related protein	-1.5
BCAL1612	Putative arginyl-tRNA:protein arginylyltransferase	-2.3
BCAL2442	Heat shock protein 90	-1.8
BCAL3146/groEL	Chaperonin GroEL	-3.8
BCAL3147/groES	Co-chaperonin GroES	-4.5
BCAM0548	Chaperonin GroEL	-3.1
BCAM0549	Co-chaperonin GroES	-3.2
BCAM0548	Chaperonin GroEL	-3.1
BCAM1407	Probable cysteine-peptidase of the DJ-1/PfpI family	-1.8
BCAM2378/pepX	x-prolyl-dipeptidyl aminopeptidase	-1.8
BCAM2477	Serine peptidase, family S10	-3.1
BCAM2478	Serine-carboxyl peptidase	-2.5
BCAS0637/groEL	Chaperonin GroEL	-27.5
BCAS0638/groES	Co-chaperonin GroES	-29.4
Protein secretion		
Ion homeostasis		
BCAL0116	Putative TonB-dependent siderophore receptor; similar to ferric coprogen and ferric-rhodotorulic acid	-1.5
BCAL0207	Putative transcriptional regulator of the AsnC family	-2.8
BCAL3297	Putative ferritin DPS-family DNA binding protein	-3.5
BCAM0491	TonB-dependent receptor similar to ferric coprogen and ferric-rhodotorulic acid	-1.8
BCAM2227/phcG	Putative pyochelin biosynthetic protein PchG	-1.6
BCAM2230/phcE	Dihydroaeruginoic acid synthetase PchE involved in pyochelin biosynthesis	-1.5

Replication, transcription and transcriptional regulation

Transcriptional regulators

BCAL0865	RpiR family regulatory protein	-1.5
BCAL0054	Putative transcriptional regulator of the MerR family	-1.5
BCAL0812	sigma-54 modulation protein	-6.9
BCAL0867	Endoribonuclease	-1.5
BCAL0813/rpoN	Putative RNA polymerase sigma-54 factor	-1.5
BCAL1217	Transcriptional regulator of the LysR family	-1.6
BCAL1230	Transcriptional regulator of the LysR family	-1.6
BCAL1274	Putative transcriptional regulator predicted to have a role in phosphate uptake; similar to PhoU	-1.6
BCAL1403	Transcriptional regulator of the LysR family	-1.5
BCAL1637	Putative DNA-binding protein	-1.6
BCAL2309	Putative transcriptional regulator of the MerR family	-1.6
BCAL2423	Transcription factor of the TetR family	-2.2
BCAL2443	Transcription factor of the GntR family	-1.6
BCAL2812	Putative Fur family transcriptional regulator	-1.7
BCAL3382	Transcription factor of the GntR family	-1.6
BCAM0189	Transcriptional regulator of the AraC family	-2.6
BCAM0530	Transcription factor of the GntR family	-2.1
BCAM0635	Transcription factor of the AnsC family	-1.6
BCAM0966	Transcription factor of the GntR family	-1.7
BCAM1139	Transcription factor of the MarR family	-1.9
BCAM1406	Transcription factor of the LacI family	-1.6
BCAM1483	Cyclic nucleotide-binding transcriptional regulator	-1.6
BCAM1781	Transcriptional regulator of the LysR family	-1.5
BCAM1966	Transcriptional regulator of the ArsR family	-2.3
BCAM2019	Transcriptional regulator of the AraC family	-1.6
BCAM2059	LuxR superfamily regulatory protein	-2.5
BCAM2480	Transcription factor of the AnsC family	-1.6
BCAM2569	Transcription factor of the IciR family	-2.0
BCAM2652	Transcription factor of the TetR family	-1.8
BCAM2708	Transcription factor of the IciR family	-1.9
BCAM2777	Transcriptional regulator of the LysR family	-1.6

BCAS0258	Transcription factor of the GntR family	-1.7
BCAS0603	Transcriptional regulator of the AraC family	-1.5
BCAS0736	Transcription factor of the TetR family	-1.8
BCAS0761	Transcription factor of the AraC family	-1.6
BCAS0770	Transcription factor of the LysR family	-1.5
BCAL0609	transcriptional regulator-like	-2.3
BCAL02872	sigma factor 70 EcfA2	-2.2
BCAL1810	Transcription factor of the TetR family	-2.3
DNA repair		
BCAL0437	O6-methylguanine-DNA methyltransferase involved in DNA repair	-1.6
Transcription		
BCAM0580	RNase II stability modulator	-1.6
BCAM1351	Putative regulatory protein similar to RNA polymerase-binding protein DksA	-2.9
Recombination		
BCAM1953	Putative transposase	-1.5
BCAL2773/xerD	site-specific tyrosine recombinase XerD	-1.6
BCAL1142	Transposase	-1.6
BCAL1096	Putative exodeoxyribonuclease V gamma chain involved in DNA recombination	-1.5
Others		
BCAL0460	DNA topoisomerase III	-1.5
BCAL3427/hctB	Histone H1-like protein	-4.7
BCAM0004/parB	Chromosome partitioning protein ParB	-1.7
BCAM1012	Putative histone-like protein	-2.0
BCAM2179	Putative ribonuclease	-1.95
BCAM2711	H-NS histone family protein	-1.73
BCAS0191	Putative endoribonuclease	-1.87

Protein trafficking and secretion		
BCAM2040/bcscQ	Type III secretion system protein	-1.7
BCAM2041/bcscR	Type III secretion system protein	-1.7
BCAM2042/virB1	Type III secretion system protein	-1.7
BCAM2048/bcscN	Type III secretion system protein	-1.7
BCAM2050/bcscK	Type III secretion system protein	-1.7
BCAM2051/bcscJ	Type III secretion system protein	-1.7
BCAM2052	Protein of unknown function of the type III secretion system	-1.7
BCAM2053	Protein of unknown function of the type III secretion system	-2.8
BCAM2055/bcscC	Type III secretion system protein	-1.8
BCAM2056/bcscS	Type III secretion system protein	-1.8
BCAL0341	Putative type VI secretion system protein TssB	-1.6
BCAL0344	Putative type VI secretion system protein TssE	-1.5
BCAL1522	Putative exported heme utilisation related protein; predicted to be involved in hemolysin activation/secretion protein	-1.8
Signalling		
BCAL0534	Two-component regulatory system, response regulator protein	-1.6
BCAL0535	Sensor kinase protein	-2.0
BCAL1275/phoB	Phosphate regulon two-component regulatory system, response regulator protein	-2.6
BCAL1276	Putative phosphate regulon two-component regulatory system, sensor kinase protein	-2.2
BCAL1663	PrkA family serine protein kinase	-1.8
BCAM0221	Two-component regulatory system, response regulator protein	-1.5
BCAM0622	Two-component regulatory system, sensor kinase protein	-1.5
BCAM0623	Two-component regulatory system, response regulator protein	-1.6
BCAM1484	Two-component regulatory system, response regulator protein	-1.6
BCAL0128	response regulator receiver protein	-2.1
Stress response		
BCAL0463	Putative thioredoxin	-1.5
BCAL0639	Putative glutathione S-transferase involved in reduced glutathione biosynthesis	-1.6
BCAL1250	Putative glutathione S-transferase involved in glutathione biosynthesis	-1.6

BCAL2013	Putative peroxiredoxin of the AhpC/TSA family	-1.9
BCAL2106/gpo	Glutathione peroxidase	-1.8
BCAL2268	Putative stress protein	-2.0
BCAL3299/katB	Peroxidase/catalase KatB	-1.6
BCAL3331	Putative glutathione S-transferase	-1.7
BCAM0050	Universal stress-related protein; related to UspA	-3.0
BCAM0507	Similar to the general stress response protein CsbD	-3.8
BCAM0590	Putative glutathione S-transferase involved in reduced glutathione biosynthesis	-2.0
BCAM1495	Putative universal stress protein; similar to UspA	-4.1
BCAM1500	Putative universal stress protein	-4.4
BCAM1829	Putative universal stress protein	-2.1
BCAM2385/arr	Rifampin ADP-ribosyl transferase	-1.7
BCAS0726	Methionine sulfoxide reductase A	-1.8
Phage-related genes		
Bcen_3131	transposase, IS4	-2.20
Unknown		
BCAL0002		-1.7
BCAL0057		-1.8
BCAL0063		-1.7
BCAL0120		-1.5
BCAL0193		-1.8
BCAL0353		-1.7
BCAL0353		-1.7
BCAL0427		-2.2
BCAL0431		-1.6
BCAL0455		-1.7
BCAL0511		-1.6
BCAL0514		-1.7
BCAL0617		-1.6
BCAL0619		-1.6
BCAL0686		-1.5
BCAL0763		-1.8

BCAL0783	-1.7
BCAL0786	-3.1
BCAL0788	-1.9
BCAL0789	-1.7
BCAL0830	-1.8
BCAL0858	-1.5
BCAL0889	-1.9
BCAL0928	-3.0
BCAL0931	-1.6
BCAL0947a	-1.8
BCAL1145	-1.6
BCAL1165	-1.9
BCAL1166	-2.0
BCAL1167	-1.7
BCAL1168	-1.7
BCAL1251	-1.7
BCAL1282	-1.6
BCAL1337	-1.6
BCAL1341	-1.5
BCAL1342	-1.5
BCAL1374	-1.6
BCAL1475	-1.8
BCAL1495	-1.6
BCAL1496	-5.4
BCAL1522	-1.8
BCAL1629	-1.7
BCAL1664	-1.7
BCAL1791	-2.2
BCAL1794	-1.7
BCAL1795	-2.0
BCAL1816	-1.5
BCAL1830	-6.2
BCAL1832	-1.6
BCAL1835	-1.5

BCAL1858	-1.6
BCAL1868	-2.1
BCAL1917	-1.7
BCAL1952	-2.9
BCAL2025	-1.5
BCAL2027	-2.6
BCAL2269	-1.9
BCAL2270	-2.0
BCAL2271	-1.8
BCAL2306	-1.6
BCAL2307	-2.2
BCAL2418	-1.9
BCAL2434	-2.0
BCAL2440	-1.6
BCAL2457	-1.7
BCAL2475a	-1.7
BCAL2512	-1.5
BCAL2635	-2.1
BCAL2684	-1.6
BCAL2745	-1.8
BCAL2763	-1.6
BCAL2786	-2.7
BCAL2828	-2.7
BCAL2980	-1.7
BCAL2998	-1.6
BCAL3003	-1.9
BCAL3018	-2.1
BCAL3170	-1.7
BCAL3192	-2.4
BCAL3211	-1.7
BCAL3298	-2.1
BCAL3321	-1.5
BCAL3352	-1.6
BCAM0006	-1.6

BCAM0013	-2.3
BCAM0015	-1.6
BCAM0025	-1.9
BCAM0030	-1.8
BCAM0031	-1.9
BCAM0042	-1.7
BCAM0052	-1.7
BCAM0053/srpH	-1.5
BCAM0165	-1.6
BCAM0190	-1.9
BCAM0338	-1.6
BCAM0339	-1.6
BCAM0444	-1.6
BCAM0493	-1.9
BCAM0529A	-1.7
BCAM0565	-1.6
BCAM0576	-1.7
BCAM0589	-2.2
BCAM0634	-2.4
BCAM0709	-1.9
BCAM0753	-2.3
BCAM0888	-1.5
BCAM0929	-1.8
BCAM0943	-2.1
BCAM1014	-2.7
BCAM1041	-1.8
BCAM1058	-1.5
BCAM1227	-1.9
BCAM1230	-1.6
BCAM1234	-1.7
BCAM1250	-2.5
BCAM1352	-2.5
BCAM1363	-1.8
BCAM1412	-2.1

BCAM1413a	-2.7
BCAM1414	-2.2
BCAM1443	-2.7
BCAM1479	-1.6
BCAM1480	-2.9
BCAM1481	-4.2
BCAM1482	-3.5
BCAM1492	-2.0
BCAM1496	-2.0
BCAM1502	-5.0
BCAM1529	-1.6
BCAM1538	-1.9
BCAM1547	-1.7
BCAM1548	-1.6
BCAM1569	-2.2
BCAM1589	-1.6
BCAM1601	-1.8
BCAM1602	-2.8
BCAM1733	-1.8
BCAM1735	-2.1
BCAM1736	-2.0
BCAM1742	-2.6
BCAM1777A	-2.1
BCAM1782	-1.8
BCAM1800	-3.3
BCAM1869	-1.6
BCAM1926	-1.9
BCAM1967/xenB	-2.2
BCAM2062	-1.7
BCAM2068	-2.3
BCAM2069	-1.9
BCAM2071	-1.9
BCAM2083	-1.6
BCAM2084	-1.8

BCAM2085	-1.6
BCAM2154	-1.7
BCAM2167	-2.3
BCAM2178	-1.6
BCAM2181	-2.3
BCAM2182	-2.1
BCAM2199	-1.5
BCAM2206	-1.8
BCAM2207	-1.6
BCAM2213	-1.6
BCAM2237	-1.6
BCAM2238	-1.8
BCAM2253A	-1.8
BCAM2254	-1.9
BCAM2287	-1.7
BCAM2308	-2.5
BCAM2309	-1.6
BCAM2336	-1.8
BCAM2338	-2.3
BCAM2339	-1.7
BCAM2357	-2.2
BCAM2377	-1.5
BCAM2379	-1.7
BCAM2402	-1.7
BCAM2403	-1.6
BCAM2405	-2.3
BCAM2413	-1.5
BCAM2487	-1.8
BCAM2504	-2.3
BCAM2518	-1.6
BCAM2555	-2.3
BCAM2595	-1.5
BCAM2625	-1.7
BCAM2642	-1.6

BCAM2657	-1.6
BCAM2690	-1.6
BCAM2705	-1.5
BCAM2706	-1.7
BCAM2713	-1.6
BCAM2731	-1.7
BCAM2740	-1.5
BCAM2741	-1.5
BCAM2742	-3.4
BCAM2755	-1.7
BCAM2834	-1.5
BCAS0016	-2.0
BCAS0098	-1.5
BCAS0154	-1.7
BCAS0155	-1.8
BCAS0159	-1.5
BCAS0194	-1.6
BCAS0246	-2.1
BCAS0260	-1.7
BCAS0292	-1.7
BCAS0319	-1.8
BCAS0331	-1.8
BCAS0371	-1.5
BCAS0406	-2.3
BCAS0407	-1.5
BCAS0636	-9.9
BCAS0640	-3.2
BCAS0760	-1.7
BCAS0762	-1.5
Bcen_2651	-2.4
BCAL0108	-2.2
Bcen_2885	-3.1
Bcen_2886	-5.3
Bcen_2900	-2.1

BCAL0436		-1.6
BCAM2163		-2.0
BCAM2350	ATPase component of a Putative ABC transporter	-1.5
BCAM2351	Permease component of a Putative ABC transporter	-1.5
BCAM0505	Putative membrane-attached protein	-2.1

Genes down-regulated in IST4113, compared to IST439, sorted by gene number

BCAL0002
BCAL0024
BCAL0042
BCAL0046
BCAL0047
BCAL0054
BCAL0057
BCAL0063
BCAL0064
BCAL0067
BCAL0068
BCAL0108
BCAL0116
BCAL0120
BCAL0120
BCAL0124
BCAL0126
BCAL0127
BCAL0128
BCAL0128
BCAL0131
BCAL0136
BCAL0193
BCAL0207
BCAL0212
BCAL0213
BCAL0282
BCAL0283
BCAL02872
BCAL0317
BCAL0319

BCAL0328
BCAL0329
BCAL0330
BCAL0341
BCAL0344
BCAL0353
BCAL0353
BCAL0388
BCAL0398
BCAL0404
BCAL0405
BCAL0406
BCAL0407
BCAL0408
BCAL0427
BCAL0431
BCAL0436
BCAL0437
BCAL0455
BCAL0460
BCAL0460
BCAL0463
BCAL0476
BCAL0480
BCAL0503
BCAL0511
BCAL0514
BCAL0534
BCAL0535
BCAL0536
BCAL0540
BCAL0543
BCAL0609
BCAL0617

BCAL0619
BCAL0639
BCAL0686
BCAL0763
BCAL0783
BCAL0784
BCAL0785
BCAL0786
BCAL0788
BCAL0789
BCAL0812
BCAL0813
BCAL0830
BCAL0858
BCAL0865
BCAL0867
BCAL0889
BCAL0928
BCAL0931
BCAL0947a
BCAL1046
BCAL1047
BCAL1076
BCAL1096
BCAL1142
BCAL1145
BCAL1165
BCAL1166
BCAL1167
BCAL1168
BCAL1217
BCAL1230
BCAL1233
BCAL1247

BCAL1249
BCAL1250
BCAL1251
BCAL1271
BCAL1272
BCAL1273
BCAL1274
BCAL1275
BCAL1276
BCAL1282
BCAL1337
BCAL1341
BCAL1342
BCAL1374
BCAL1390
BCAL1400
BCAL1403
BCAL1418
BCAL1472
BCAL1473
BCAL1475
BCAL1495
BCAL1496
BCAL1510
BCAL1511
BCAL1522
BCAL1522
BCAL1612
BCAL1629
BCAL1637
BCAL1647
BCAL1663
BCAL1664
BCAL1677

BCAL1791
BCAL1794
BCAL1795
BCAL1810
BCAL1816
BCAL1829
BCAL1830
BCAL1832
BCAL1835
BCAL1858
BCAL1868
BCAL1917
BCAL1952
BCAL1956
BCAL1975
BCAL2013
BCAL2024
BCAL2025
BCAL2027
BCAL2041
BCAL2106
BCAL2141
BCAL2142
BCAL2143
BCAL2144
BCAL2205
BCAL2206
BCAL2268
BCAL2269
BCAL2270
BCAL2271
BCAL2305
BCAL2306
BCAL2307

BCAL2309
BCAL2352
BCAL2418
BCAL2423
BCAL2434
BCAL2440
BCAL2441
BCAL2442
BCAL2443
BCAL2457
BCAL2472
BCAL2475a
BCAL2512
BCAL2635
BCAL2636
BCAL2645
BCAL2680
BCAL2681
BCAL2682
BCAL2683
BCAL2684
BCAL2690
BCAL2745
BCAL2746
BCAL2763
BCAL2773
BCAL2783
BCAL2786
BCAL2791
BCAL2792
BCAL2812
BCAL2814
BCAL2828
BCAL2829

BCAL2899
BCAL2958
BCAL2980
BCAL2998
BCAL3003
BCAL3008
BCAL3018
BCAL3107
BCAL3110
BCAL3111
BCAL3146
BCAL3147
BCAL3166
BCAL3170
BCAL3191
BCAL3192
BCAL3211
BCAL3276
BCAL3296
BCAL3297
BCAL3298
BCAL3299
BCAL3321
BCAL3331
BCAL3352
BCAL3359
BCAL3379
BCAL3380
BCAL3381
BCAL3382
BCAL3390
BCAL3427
BCAL3428
BCAL3473

BCALr1625
BCAM0004
BCAM0006
BCAM0013
BCAM0015
BCAM0023
BCAM0025
BCAM0030
BCAM0031
BCAM0042
BCAM0050
BCAM0052
BCAM0053
BCAM0086
BCAM0096
BCAM0138
BCAM0165
BCAM0186
BCAM0189
BCAM0190
BCAM0221
BCAM0224
BCAM0254
BCAM0338
BCAM0339
BCAM0384
BCAM0408
BCAM0416
BCAM0444
BCAM0491
BCAM0493
BCAM0505
BCAM0507
BCAM0529A

BCAM0530
BCAM0548
BCAM0548
BCAM0549
BCAM0565
BCAM0576
BCAM0580
BCAM0584
BCAM0589
BCAM0590
BCAM0592
BCAM0622
BCAM0623
BCAM0634
BCAM0635
BCAM0709
BCAM0753
BCAM0774
BCAM0888
BCAM0929
BCAM0943
BCAM0946
BCAM0952
BCAM0954
BCAM0962
BCAM0964
BCAM0965
BCAM0966
BCAM1012
BCAM1014
BCAM1015
BCAM1041
BCAM1058
BCAM1111

BCAM1112
BCAM1139
BCAM1227
BCAM1230
BCAM1234
BCAM1250
BCAM1351
BCAM1352
BCAM1353
BCAM1361
BCAM1363
BCAM1406
BCAM1407
BCAM1412
BCAM1413a
BCAM1414
BCAM1430
BCAM1443
BCAM1477
BCAM1479
BCAM1480
BCAM1481
BCAM1482
BCAM1483
BCAM1484
BCAM1492
BCAM1495
BCAM1496
BCAM1500
BCAM1502
BCAM1529
BCAM1538
BCAM1547
BCAM1548

BCAM1550
BCAM1569
BCAM1570
BCAM1589
BCAM1598
BCAM1601
BCAM1602
BCAM1733
BCAM1734
BCAM1734
BCAM1735
BCAM1736
BCAM1737
BCAM1738
BCAM1740
BCAM1742
BCAM1745
BCAM1777A
BCAM1781
BCAM1782
BCAM1787
BCAM1800
BCAM1829
BCAM1833
BCAM1869
BCAM1926
BCAM1931
BCAM1953
BCAM1966
BCAM1967/xenB
BCAM2006
BCAM2019
BCAM2040
BCAM2041

BCAM2042
BCAM2048
BCAM2050
BCAM2051
BCAM2052
BCAM2053
BCAM2055
BCAM2056
BCAM2059
BCAM2062
BCAM2067
BCAM2068
BCAM2069
BCAM2071
BCAM2080
BCAM2083
BCAM2084
BCAM2085
BCAM2143
BCAM2154
BCAM2163
BCAM2167
BCAM2169
BCAM2176
BCAM2178
BCAM2179
BCAM2181
BCAM2182
BCAM2199
BCAM2206
BCAM2207
BCAM2208
BCAM2213
BCAM2227

BCAM2230
BCAM2237
BCAM2238
BCAM2253A
BCAM2254
BCAM2287
BCAM2307
BCAM2308
BCAM2309
BCAM2319
BCAM2325
BCAM2326
BCAM2336
BCAM2337
BCAM2338
BCAM2339
BCAM2340
BCAM2343
BCAM2350
BCAM2351
BCAM2357
BCAM2377
BCAM2378
BCAM2379
BCAM2381
BCAM2383
BCAM2385
BCAM2392
BCAM2402
BCAM2403
BCAM2405
BCAM2413
BCAM2430
BCAM2431

BCAM2432
BCAM2433
BCAM2469
BCAM2477
BCAM2478
BCAM2480
BCAM2487
BCAM2504
BCAM2518
BCAM2555
BCAM2556
BCAM2568
BCAM2569
BCAM2590
BCAM2591
BCAM2595
BCAM2607
BCAM2625
BCAM2642
BCAM2652
BCAM2657
BCAM2690
BCAM2705
BCAM2706
BCAM2708
BCAM2710
BCAM2711
BCAM2713
BCAM2731
BCAM2740
BCAM2741
BCAM2742
BCAM2755
BCAM2777

BCAM2791
BCAM2822
BCAM2834
BCAM505
BCAS0016
BCAS0017
BCAS0023
BCAS0034
BCAS0098
BCAS0154
BCAS0155
BCAS0159
BCAS0181
BCAS0191
BCAS0194
BCAS0230
BCAS0236
BCAS0240
BCAS0246
BCAS0253
BCAS0258
BCAS0259
BCAS0260
BCAS0292
BCAS0292
BCAS0293
BCAS0319
BCAS0320
BCAS0331
BCAS0371
BCAS0406
BCAS0407
BCAS0409
BCAS0460

BCAS0603
BCAS0636
BCAS0637
BCAS0638
BCAS0640
BCAS0726
BCAS0736
BCAS0760
BCAS0761
BCAS0762
BCAS0770
Bcen_2651
Bcen_2885
Bcen_2886
Bcen_2900
Bcen_3131
