# Supplementary material for: Genomic Expression Analysis Reveals Strategies of Burkholderia cenocepacia to Adapt to Cystic Fibrosis Patients' Airways and Antimicrobial Therapy
Source: PLoS One. 2011 Dec 21;6(12):e28831. doi: 10.1371/journal.pone.0028831 (PMC3244429; doi:10.1371/journal.pone.0028831)
Supplement: Table S4 — List of genes having an altered expression in B. cenocepacia variants IST439 and IST4113 that are documented to be regulated by CciR. The microarray dataset was searched for genes documented to be activated or repressed by the CciR transcription factor using the results of O'Grady et al., (2010). (PDF) [file pone.0028831.s004.pdf]

**Table S3 - List of genes having an altered expression in IST439 and IST4113 that are documented to be regulated by CciR.** The microarray dataset was searched for genes documented to be activated or repressed by the CciR transcription factor using the results of O'Grady et al., (2010).

**Genes up-regulated in IST4113 and documented to be activated by CciR**

| | |
|---|---|
| BCAL0683 | BCAL0140 |
| BCAL0110 | BCAL0683 |
| BCAL0144 | BCAL0151 |
| BCAL0425 | BCAL1742 |
| BCAL0600 | |
| BCAL0778 | BCAL2734 |
| BCAL0779 | BCAM0153 |
| BCAL0780 | BCAM0166 |
| BCAL1671 | BCAM0693 |
| BCAL1944 | BCAM0705 |
| BCAL2354 | BCAM1305 |
| BCAL2715 | BCAM1700 |
| BCAM1377 | BCAM2589 |
| BCAM1427 | BCAM2674 |
| BCAM1588 | BCAS0110 |
| BCAM2545 | BCAS0383 |
| BCAS0138 | BCAS0387 |
| BCAS0386 | BCAS0388 |
| BCAS0397 | BCAS0396 |

**Genes down-regulated in IST4113 and documented to be repressed by CciR**

| | | | | |
|---|---|---|---|---|
| BCAM0186 | BCAL0124 | BCAM0339 | BCAM1966 | BCAM2469 |
| BCAS0409 | BCAL0283 | BCAM0529A | BCAM2053 | BCAM2568 |
| BCAL0064 | BCAL1145 | BCAM0634 | BCAM2062 | BCAM2731 |
| BCAL0437 | BCAL1677 | BCAM0774 | BCAM2287 | BCAS0638 |
| BCAL2635 | BCAL1952 | BCAM0965 | BCAM2307 | |
| BCAL2636 | BCAL2268 | BCAM1111 | BCAM2319 | |
| BCAL2828 | BCAL2270 | BCAM1112 | BCAM2325 | |
| BCAL2829 | BCAL2352 | BCAM1250 | BCAM2377 | |
| BCAM0943 | BCAL2457 | BCAM1412 | BCAM2379 | |
| BCAM1733 | BCAL2472 | BCAM1734 | BCAM2430 | |
| BCAM2167 | BCAL3427 | BCAM1742 | BCAM2431 | |
| BCAM2652 | BCAM0013 | BCAM1800 | BCAM2432 | |
