# Supplementary material for: Genomic Expression Analysis Reveals Strategies of Burkholderia cenocepacia to Adapt to Cystic Fibrosis Patients' Airways and Antimicrobial Therapy
Source: PLoS One. 2011 Dec 21;6(12):e28831. doi: 10.1371/journal.pone.0028831 (PMC3244429; doi:10.1371/journal.pone.0028831)
Supplement: Table S5 — Proteins found in the membrane-enriched fraction and in the cytosolic fraction whose content was increased (above 1.5-fold) or decreased (below 0.7-fold) in the proteome of B. cenocepacia IST4113 cells, compared to the values registered in B. cenocepacia IST439 (Madeira et al ., 2011). The genes predicted to encode these proteins are also indicated and those found to be differently expressed in the two variants, based on the results of the microarray analysis carried out in this study, are highlighted in bold. (PDF) [file pone.0028831.s005.pdf]

Table S5. Proteins found in the membrane-enriched fraction and in the cytosolic fraction whose content was increased (above 1.5-fold) or decreased (below 0.7-fold) in the proteome of IST4113 cells, compared to the values registered in IST439 (Madeira et al., 2011). The genes predicted to encode these proteins are also indicated and those found to be differently expressed in the two variants, based on the results of the microarray analysis carried out in this study, are highlighted in bold.

**Spot (nr.)**	**Protein Function (Gene name)**	**Fold Change Protein content (IST4113/IST439**	**Fold Change Gene Expression (IST4113/IST439**
	**Amino acid metabolism and transport**		
16	Oligopeptidase A, Zn-dependent oligopeptidases (BCAL2213)	2.5	-
36	Acetylglutamate kinase (ArgB; BCAL0496)	1.6	-
37		2.4	
52	ATP phosphoribosyltransferase (HisG; BCAL0311)	2.2	-
90	**Saccharopine dehydrogenase (Lys1; BCAL1796)**	**0.7**	**1.5**
96		0.7	
143	Serine-pyruvate aminotransferase (BCAM0512)	2.5	
153	Histidinol dehydrogenase (HisD; BCAL0312)	1.5	-
	**Cell envelope biogenesis, outer membrane and lipid metabolism**		
15	**Acetyl/propionyl-CoA carboxylase, alpha subunit (BCAM2430)**	**2.0**	**0.6**
35	D-alanine-D-alanine ligase (Ddl; BCAL3460)	2.0	-
44	**Enoyl-CoA hydratase/carnithine racemase (BCAL0409)**	**0.7**	**0.5**
68	FAD-binding 9, Siderophore-interacting protein (BCAM0026)	3.2	-
149	**Cyclopropane fatty acid synthase (BCAL2783)**	**3.1**	**0.5**
156	Acyl-coenzyme A synthetases/AMP-(fatty) acid ligase/acetyl-CoA synthetase (BCAL2284)	1.9	-
167	Acetyl-CoA carboxylase biotin carboxyl carrier protein subunit (AccA)(BCAL3420)	0.6	-
198	(3R)-hydroxymyristoyl-(acyl carrier protein) dehydratase (FabZ)	0.7	-
	**Energy production and conversion**		
9	Pyruvate dehydrogenase complex, dehydrogenase (E1) component (AceE)	3.5	-

**Spot (nr.)**	**Protein Function (Gene name)**	**Fold Change Protein content (IST4113/IST439**	**Fold Change Gene Expression (IST4113/IST439**
155		3.4	-
160		3.4	-
10	Aconitate hydratase (AcnA)(BCAM2701)	3.0	-
13	Transketolase (Tkt)(BCAL3389)	1.5	-
38	Electron transfer flavoprotein, alpha subunit (FixB)(BCAL2934)	1.6	-
47	2-keto-3-deoxy-6-phosphogluconate aldolase (Eda)(BCAL3366)	1.5	-
81	**Aldo/keto reductase (Tas)(BCAM0042)**	**2.3**	**0.6**
92	Fructose/tagatose bisphosphate aldolase (Fba)(BCAL2839)	2.1	-
108	Citrate synthase (GltA)(BCAM0972)	2.3	-
109	Dihydrolipoamide dehydrogenase (LpdA)(BCAL1517)	3.0	-
189		1.7	-
112	NAD-dependent aldehyde dehydrogenase (BCAM2468)	3.4	-
132	NADH dehydrogenase/NADH:ubiquinone oxidoreductase 75 kD (NuoG)(BCAL2338)	3.6	-
147	Phosphopyruvate hydratase (Eno)(BCAL2179)	0.7	-
168	Malate synthase G (AceB)(BCAM2821)	2.6	-
170	Phosphoenolpyruvate carboxykinase (PckG)(BCAM1581)	3.8	-
171		3.9	-
	**Nucleotide transport and metabolism**		
3	Carbamoyl phosphate synthase large subunit (CarB)(BCAL1262)	2.2	-
33	Phosphoribosylaminoimidazole (AIR) synthetase (PurM)(BCAL3162)	2.3	-
105	Adenylosuccinate synthetase (PurA)(BCAL1873)	0.7	-
113	Bifunctional phosphoribosylaminoimidazolecarboxamide formyltransferase/IMP cyclohydrolase (PurH)(BCAL3336)	5.2	-
115	**Bifunctional GMP synthase/Glutamine amidotransferase protein (GuaA)(BCAL2061)**	**1.5**	**1.7**
121	**IMP dehydrogenase/GMP reductase (GuaB)(BCAL2063)**	**3.2**	**1.5**
	**Posttranslational modification, protein turnover, chaperones**		
2	Molecular chaperone DnaK (DnaK)(BCAL3270)	2.0	-
154		2.1	-
8	AAA ATPase (ClpB)(BCAL1919)	6.0	-

Spot (nr.)	Protein Function (Gene name)	Fold Change Protein content (IST4113/IST439	Fold Change Gene Expression (IST4113/IST439
159		5.4	-
29	ATP-dependent Clp protease proteolytic subunit (ClpP)(BCAL1996)	0.7	-
130	**60 kDa chaperonin (GroEL)(BCAL3146)**	**1.7**	**0.3**
176	**Trigger factor (Tig)(BCAL1997)**	**2.6**	**1.5**
	**Secondary metabolites biosynthesis, transport and catabolism**		
34	**Acetoacetate decarboxylase (Adc)(BCAM0023)**	**2.1**	**0.6**
64	3-hydroxybutyrate dehydrogenase (Bdh)(BCAM0022)	2.3	-
	**Translation**		
4	Leucyl-tRNA synthetase (LeuS)(BCAL3373)	3.4	-
7	Phenylalanyl-tRNA synthetase (PheT)(BCAL1486)	3.6	-
17	**Ribosomal protein S1 (RpsA)**	**2.7**	**1.7**
18		2.3	-
177		2.4	-
22	**Elongation factor Tu (Tuf)**	**2.9**	**1.8**
178	Alanyl-tRNA synthetase (AlaS)(BCAL1416)	2.9	-
181	Arginyl-tRNA synthetase (ArgS)(BCAL0679)	2.9	-
183	Aspartyl/glutamyl-tRNA amidotransferase subunit A (GatA) (BCAL0484)	4.1	-
186	**Elongation factor G (FusA)(BCAL0231)**	**2.5**	**2.5**
187	Isoleucyl-tRNA synthetase (IleS)(BCAL2724)	2.5	-
190	Elongation factor Ts (Tsf)(BCAL2090)	2.3	-
	**Other functions**		
	Defense mechanisms		
75	Carboxylesterase (BCAL2816)	1.6	-
	Coenzyme metabolism		
20	S-adenosylmethionine synthetase (MetK)(BCAL0509)	0.7	-
69	Methenyltetrahydrofolate cyclohydrolase (FolD)(BCAL2212)	1.6	-
	Antioxidants		
201	Putative hydroperoxide reductase (AhpC)	0.7	-
	**Unknown function**		
128	**Conserved hypothetical protein (BCAS0291)**	**3.0**	**0.1**

Spot (nr.)	Protein Function (Gene name)	Fold Change Protein content (IST4113/IST439	Fold Change Gene Expression (IST4113/IST439
131	**Conserved hypothetical protein (BCAS0292)**	**0.7**	**0.7**

PROTEINS FOUND IN THE MEMBRANE-ENRICHED FRACTION

Spot (nr.)	Protein description(Gene name)	Fold Change Protein content (IST4113/IST439	Fold Change Gene Expression (IST4113/IST439
	**Amino acid metabolism and transport**		
231	**ABC transporter (LivF)(BCAM2247)**	**1.5**	**2.1**
255	5,10-methylenetetrahydrofolate reductase (MetF)(BCAL0147)	0.5	-
270	Glycine/serine hydroxymethyltransferase (GlyA)(BCAL3197)	0.7	-
277	Lysine decarboxylase (BCAL2641)	0.4	-
	**Cell envelope biogenesis, outer membrane and lipid metabolism**		
201	Outer membrane protein/protective antigen (BCAL2083)	4.7	-
267	Phosphomannomutase (BCAL3113)	0.7	-
269	NAD-dependent epimerase/dehydratase (BCAL1071)	0.6	-
	**Energy production and conversion**		
205	Succinate dehydrogenase flavoprotein subunit (SdhA)(BCAM0969)	1.6	-
227	F0F1-type ATP synthase, alpha subunit (AtpA)(BCAL0034)	1.8	-
229	**Isocitrate lyase (AceA)(BCAM1588)**	**1.5**	**2.3**
254	Transketolase (Tkt)(BCAL3389)	0.5	-
257	Electron transfer flavoprotein, alpha subunit (FixB)(BCAL2934)	0.5	-
261	Fumarase (FumC)(BCAL2908)	0.6	-
276	Phosphoenolpyruvate synthase (PpsA)(BCAL2074)	0.5	-
	**Adaptation to atypical conditions**		
252	PhoH-like protein (BCAL1937)	0.5	-
258	Transcription antitermination protein (NusG)(BCAL0221)	0.6	-
	**Translation**		
256	YbaK/prolyl-tRNA synthetase associated region (BCAL0630)	0.5	-

Spot (nr.)	Protein description(Gene name)	Fold Change Protein content (IST4113/IST439	Fold Change Gene Expression (IST4113/IST439
	**Inorganic ion transport and metabolism**		
202	TonB-dependent siderophore receptor (BCAM2224)	1.8	-
203	TonB-dependent copper receptor (BCAM0948)	1.8	-
	**Intracellular trafficking and secretion**		
232	Predicted periplasmic/secreted protein (BCAL2669)	1.7	-
	**Transport of small molecules**		
235	ABC transporter related (BCAM1210)	1.6	-
263	Extracellular solute-binding protein (BCAS0242)	0.6	-
